# CRISPR/Cas9-based QF2 knock-in at the *tyrosine hydroxylase* (*th*) locus reveals novel *th*-expressing neuron populations in the zebrafish mid- and hindbrain

**DOI:** 10.3389/fnana.2023.1196868

**Published:** 2023-08-02

**Authors:** Christian Altbürger, Jens Holzhauser, Wolfgang Driever

**Affiliations:** ^1^Developmental Biology, Faculty of Biology, Institute of Biology I, Albert Ludwigs University Freiburg, Freiburg, Germany; ^2^CIBSS and BIOSS - Centres for Biological Signalling Studies, Albert Ludwigs University Freiburg, Freiburg, Germany

**Keywords:** catecholamines, dopaminergic neurons, noradrenergic neurons, zebrafish, brain evolution, tyrosine hydroxylase, genome engineering

## Abstract

Catecholaminergic neuron clusters are among the most conserved neuromodulatory systems in vertebrates, yet some clusters show significant evolutionary dynamics. Because of their disease relevance, special attention has been paid to mammalian midbrain dopaminergic systems, which have important functions in motor control, reward, motivation, and cognitive function. In contrast, midbrain dopaminergic neurons in teleosts were thought to be lost secondarily. Here, we generated a CRISPR/Cas9-based knock-in transgene at the *th* locus, which allows the expression of the Q-system transcription factor QF2 linked to the Tyrosine hydroxylase open reading frame by an E2A peptide. The QF2 knock-in allele still expresses Tyrosine hydroxylase in catecholaminergic neurons. Coexpression analysis of QF2 driven expression of QUAS fluorescent reporter transgenes and of *th* mRNA and Th protein revealed that essentially all reporter expressing cells also express Th*/th*. We also observed a small group of previously unidentified cells expressing the reporter gene in the midbrain and a larger group close to the midbrain–hindbrain boundary. However, we detected no expression of the catecholaminergic markers *ddc, slc6a3*, or *dbh* in these neurons, suggesting that they are not actively transmitting catecholamines. The identified neurons in the midbrain are located in a GABAergic territory. A coexpression analysis with anatomical markers revealed that Th-expressing neurons in the midbrain are located in the tegmentum and those close to the midbrain–hindbrain boundary are located in the hindbrain. Our data suggest that zebrafish may still have some evolutionary remnants of midbrain dopaminergic neurons.

## 1. Introduction

Dopaminergic (DA), noradrenergic (NA), and adrenergic neurons are grouped into the family of catecholaminergic (CA) neurons. These neuronal populations are identified in animals by the expression of Tyrosine hydroxylase (Th), the rate-limiting enzyme in catechol biogenesis (reviewed in Flames and Hobert, 2011). The development of DA neurons, especially in the mammalian midbrain, is very well-studied because their reduction or loss is associated with Parkinson's disease and has raised great biomedical research interest (Arenas et al., 2015; Klein et al., 2019). DA neurons reside in three main nuclei in the mammalian midbrain, namely, the substantia nigra pars compacta (SNc), the ventral tegmental area (VTA), and the retrorubral field (RRF) (Dahlstroem and Fuxe, 1964; Björklund and Hökfelt, 1983). Midbrain DA neurons emerge from neural progenitor cells, which are characterized by the expression of SHH and FOXA1/2 and are located in the floor plate of the ventral midbrain during neurogenesis (Ono et al., 2007; Bonilla et al., 2008; Joksimovic et al., 2009; Blaess et al., 2011; Hayes et al., 2011; Li et al., 2015). Post-mitotic progenitors express *Nr4a2, Lmx1a, Lmx1b*, and *Pitx3*, which are continued to be expressed in mature midbrain DA neurons (Zetterstrom et al., 1997; Saucedo-Cardenas et al., 1998; Asbreuk et al., 2002; Smits et al., 2003; Andersson et al., 2006). Therefore, these genes serve as markers to identify midbrain DA neurons in combination with TH. NR4A2 is also critical for the expression of DA markers such as *Th, Slc18a2/Vmat2*, and *Slc6a3/Dat* (Smits et al., 2003). The expression of these genes in combination with *dopa decarboxylase (Ddc)* serves as a marker to identify bona fide DA neurons (Flames and Hobert, 2011).

Teleosts show both conserved and divergent anatomical locations of DA and NA neuronal groups when compared with mammals (Ekström et al., 1994; Meek, 1994; Stuesse et al., 1994; Kaslin and Panula, 2001; Rink and Wullimann, 2002; Ma, 2003; Ryu et al., 2007; Yamamoto and Vernier, 2011; Yamamoto et al., 2017). Among the conserved anatomical locations are the NA groups in the hindbrain, the retinal amacrine DA neurons, and the posterior tubercular DA neurons, which appear to be homologous to the A11 mammalian groups (Ryu et al., 2007). Divergent anatomical DA groups include subpallial and pretectal teleost DA groups, which are absent in mammals, and midbrain DA groups, which are present in mammals but not yet detected in zebrafish. Teleosts, amphibians, and birds have two TH-encoding genes, *TH1* and *TH2* (Candy and Collet, 2005; Chen et al., 2009; Filippi et al., 2010; Yamamoto et al., 2010; Yamamoto and Vernier, 2011; Xavier et al., 2017), and it has been shown that *TH2* has been secondarily lost in placental mammals (Yamamoto et al., 2010). Due to the lack of *TH2* in placental mammals, “*TH1*” is simply called “*TH*” in mammals, and in accordance with this mammalian nomenclature, zebrafish “*th1*” also came to be referred to as “*th*” (zfin.org/ZDB-GENE-990621-5). We will use the latter nomenclature throughout this article. Neither *th-* nor *th2*-expressing neuronal populations homologous to mammalian midbrain DA neurons have been described in zebrafish so far (Holzschuh et al., 2001; Rink and Wullimann, 2001, 2002; Yamamoto and Vernier, 2011). The absence of midbrain DA groups in teleosts has been interpreted as a secondary loss of such populations, given that midbrain DA neurons are found in all tetrapods, lungfish (Sarcopterygii; a sister group to the Actinopterygii; Reiner and Northcutt, 1987; Stuesse et al., 1994; Yamamoto and Vernier, 2011), and in cartilaginous fishes (rays and sharks; Meredith and Smeets, 1987; Northcutt et al., 1988; Stuesse et al., 1994).

There have been suggestions that a ventral posterior tubercular DA system may provide ascending DA function into the subpallium (Rink and Wullimann, 2001); however, this system has been shown to be homologous to the mammalian A11 DA system specified in both mammals and zebrafish by the Otp transcription factor (Ryu et al., 2007), which also provides diencephalospinal DA projections and may be involved both in sensory systems (Reinig et al., 2017) and in motor modulation (Burgess and Granato, 2007; Jay et al., 2015). The zebrafish endosubpallial DA system, based on its location and arborization pattern, has been suggested to play a similar role to the mammalian mesodiencephalic DA systems (Tay et al., 2011); however, functional studies have not been performed so far, in part due to a lack of genetic tools to study the activity of subpallial DA neurons. Therefore, the question of whether a DA system providing DA neuromodulation equivalent to the mesodiencephalic mammalian systems is active in zebrafish has remained unanswered.

In this study, we generated a transgenic knock-in of the binary Q-system transcription factor QF2 (Potter et al., 2010; Subedi et al., 2014; Riabinina et al., 2015) into the *th* gene using a strategy similar to the previously published GFP knock-in approach applied to the same locus (Li et al., 2015). We used *in vivo* imaging and immunofluorescence to characterize this knock-in line and verified that QF2-driven QUAS reporter expression occurs in the same cells that express endogenous Th. Interestingly, we detected previously undescribed neuronal populations in the larval midbrain and close to the midbrain–hindbrain boundary that expresses fluorescent QUAS reporters driven by the *th:th-QF2* knock-in. To characterize these neurons, we performed fluorescent *in situ* hybridization (FISH) and hybridization chain reaction (HCR) RNA-FISH to detect low levels of transcripts (Choi et al., 2018). We found these neurons to be truly expressing *th* and not to be ectopically expressing QF2 due to reporter transgene integration site positional effects. However, analysis of additional catecholaminergic markers suggests that midbrain *th*-positive neurons may not actively transmit catecholamines but may be evolutionary remnants of a mesodiencephalic DA population.

## 2. Materials and methods

### 2.1. Zebrafish strains and animal husbandry

Zebrafish breeding and care were performed as previously described (Westerfield, 1993). Embryos were kept in the dark at 28.5°C and raised in 1x E3 medium (5 mM NaCl, 0.17 mM KCl, 0.33 mM CaCl_2_, 0.33 mM MgSO_4_) with 0.2 mM phenylthiourea to prevent pigmentation. The staging was performed as previously described (Kimmel et al., 1995). The following transgenic lines were used in this study: *th* ^*m1512Tg*^ and *th* ^*m*1513*Tg*^ (this study); *Tg(QUASr:GFP)c403* (Subedi et al., 2014); *Tg(QUAS:nlsEGFP, myl7:TagRFP)m1523* (Altbürger and Driever, unpublished).

### 2.2. Generation of the *th ^**m*1512*Tg**^* transgenic line

To generate a QF2-driver line for *th*, a previously reported knock-in technique was adapted (Li et al., 2015). Specifically, the plasmid th-P2A-Gal4 donor (Addgene #65563) was digested with BamHI and AgeI to excise the coding sequence for P2A-Gal4. The coding sequence for E2A-QF2 was amplified from the plasmid pBait-E2A-QF2 (as reported below) with the following primers: QF2 fwd2—TTCTCACAGATGCCCTGAATGTGTTGGCTGGATCCGGACAGTGTACTAATTATGCTCTCTTGAAATTGG; QF2 rev2—AAGCATAGAGGAATGATTAAGCAGAATTAATCACTGTTCGTATGTATTAATGTCGGAG. Using the NEBuilder^®^ HiFi DNA Assembly Cloning Kit, all the fragments were assembled. The plasmid pBait-E2A-QF2 was generated by (1) digestion of p3E-polyA (Kwan et al., 2007) with EcoRI and BamHI; (2) amplification of the eGFPbait + E2A sequence from the plasmid eGFPbait-E2A-KalTA4 donor (Auer et al., 2014) using the following primers: eGFPbait + E2A fwd—TAAGCTCGGGCCCTGCAGCTCTAGAGCTCGATAGTGGTACCATGGTGAGCAAG; eGFPbait + E2A rev—GCGCTTGGGTGGCATGGGACCTGGGTTGCTCTC; and (3) amplification of the QF2 sequence from the plasmid pME-QF2 (Addgene #83307) and primers QF2 fwd—AGCAACCCAGGTCCCATGCCACCCAAGCGCAAAAC; QF2 rev—CAAACTCATCAATGTATCTTATCATGTCTGTCACTGTTCGTATGTATTAATGTCGGA. All three fragments were assembled using the NEBuilder^®^ HiFi DNA Assembly Cloning Kit. To generate the *th* sgRNA, the plasmid pT7-th-sgRNA (Li et al., 2015) was linearized with HindIII, and the sgRNA was transcribed using the MEGAscript^TM^ T7 Kit, followed by ammonium acetate precipitation. For Cas9 mRNA transcription, the plasmid pCS2+hSpCas9 (Addgene #51815) (Ansai and Kinoshita, 2014) was linearized using NotI, and mRNA was transcribed using the mMESSAGE mMACHINE^TM^ SP6 Transcription Kit. To generate the knock-in of QF2 into the *th* locus, Cas9 mRNA, *th* sgRNA, and the th-E2A-QF2 plasmid were injected into single-cell stage embryos of an outcross of *Tg(QUASr:GFP)c403* (Subedi et al., 2014) fish to ABTL. GFP-positive embryos were raised to adulthood and outcrossed to ABTL fish. GFP-positive F1 embryos from three independent G0 founders were raised to adulthood and used for the generation of stable F2 generations, of which *th* ^*m1512Tg*(2*A*−*QF*2)^ was used in this study. All three alleles show Mendelian segregation. The knock-in approach relies on non-homologous end-joining (NHEJ) and is expected to integrate the eGFPbait + E2A plasmid into the *th* locus (Li et al., 2015). The *th* ^*m1512Tg*^ was not confirmed by sequencing of the locus, given that the functional QF2 protein indicates that the knock-in occurred in frame to generate the Th-E2A-QF2 open reading frame.

### 2.3. Whole mount immunofluorescence staining of larvae

Whole-mount immunofluorescence staining of larvae at 96 or 120 hpf stages was performed as previously described (Ronneberger et al., 2012). The following primary antibodies were used in this study at the indicated concentrations: chicken anti-GFP polyclonal (Thermo Fisher Scientific, #A-10262; 1:400); mouse anti-GFP monoclonal Living Colors JL-8 (ClonTech/Takara Bio USA, Inc., #632381; 1:400); rabbit polyclonal anti-Th directed against the polypeptide encoded by bp 274-1,097 of the *th* ENSEMBL transcript XM683987 (Ryu et al., 2007; Kastenhuber et al., 2010) (1:550), which does not crossreact with neurons exclusively expressing Th2 protein (Figures 4, S2, and S3 in Filippi et al., 2010); and rabbit anti-Gad1b/2 polyclonal (Abcam, ab11070; 1:500). The following secondary antibodies were used at a concentration of 1:1,000: goat anti-chicken IgY Alexa 488 (Thermo Fisher Scientific, #A-11039); goat anti-mouse IgG Alexa 488 (Thermo Fisher Scientific, #A-11001); and goat anti-rabbit IgG Alexa 555 (Thermo Fisher Scientific, #A-21430). Nuclei were stained using TOTO-3 as described before (Ronneberger et al., 2012). The immunofluorescence stainings were recorded using either an upright or inverted Zeiss LSM 880 system with the following lens: LD-LCI Plan Apochromat 25x/0.8 DIC. The histograms were adjusted linearly with ZEN. Lateral xz-views of dorsal *z*-stacks were created using the Reslice function in FIJI.

### 2.4. Whole-mount immunofluorescence staining on 30-dpf brains

The heads of 30 dpf fish were fixed in 4% PFA overnight at 4°C. The fixation was stopped with several PBST (137 mM NaCl, 2.7 mM KCl, 10 mM Na_2_HPO_4_, 1.8 mM KH_2_PO_4_, 0.1% Tween-20) washes, and the brains were dissected in PBST. After dehydration through subsequent washes in increasing concentrations of MeOH (25, 50, and 75% MeOH in PBST), the brains were stored in 100% MeOH at −20°C. For immunofluorescence staining, the brains were rehydrated and washed three times with PBST. Subsequently, the brains were incubated in Solution 1.1 {10% THEED [aminoalcohol N,N,N′,N′-Tetrakis(2-hydroxyethyl)ethylenediamine; Sigma-Aldrich, 87600-100ML], 5% Triton X-100, and 5% urea in dH_2_O (Pende et al., 2020)} overnight at 37°C. After several washes with PBST, the brains were blocked with 5% goat serum and 1% BSA in PBSTD (PBST with 1% DMSO) for 5 h at room temperature. The primary antibodies (chicken anti-GFP 1:400 and rabbit anti-Th 1:550, as detailed above) were applied in blocking solution (as detailed above) and incubated overnight at 4°C. After several washes with PBSTD, secondary antibodies (goat anti-chicken Alexa 488 1:1,000 and goat anti-rabbit Alexa 555 1:1,000) were applied in 1% blocking reagent (Roche, 11096176001) in PBSTD and incubated overnight at 4°C. Secondary antibodies were washed out with four washes of PBSTD and four washes of PBST. The stained brains were stored in 80% glycerol in PBST at 4°C. The brains were mounted in 1% agarose in 80% glycerol in glass-bottomed Petri dishes (MatTek, P35G-1.5-20-C) and recorded using an inverted Zeiss LSM 880 system with the following lens: LD-LCI Plan Apochromat 25x/0.8 DIC. The histograms were adjusted linearly with ZEN Black.

### 2.5. Whole-mount fluorescent *in situ* hybridization

Whole mount FISH was performed on the dissected brains of larvae fixed at 120 hpf with 4% PFA overnight at 4°C. The staining procedure and the combination of fluorescent *in situ* hybridization with immunofluorescence staining were performed as previously described (Ronneberger et al., 2012), except that the permeabilization was achieved through 1 h incubation in 4% Triton X-100 in PBST instead of Proteinase K treatment. The following probes were used: *ddc* (Filippi et al., 2012); *en1a* (Ekker et al., 1992); *en2a* (Ekker et al., 1992); *nr4a2a* (Filippi et al., 2007); *nr4a2b* (Filippi et al., 2007); *pax2a* (Krauss et al., 1991a); *pitx3* (Dutta et al., 2005); *slc6a3* (Holzschuh et al., 2001); *slc18a2* (Schredelseker et al., 2020). The whole mount fluorescent *in situ* hybridization coupled with immunofluorescence staining was recorded using either an upright or inverted Zeiss LSM 880 system with the following lenses: LD-LCI Plan Apochromat 25x/0.8 DIC or LD-LCI Plan Apochromat 40x/1,2 autocorr. The histograms were adjusted linearly with ZEN Black.

### 2.6. *In vivo* imaging of *th ^**m*1512*tg**^*; *Tg(QUASr:GFP)c403*

The larvae at 120 hpf or 15 dpf were anesthetized with 612 μM tricaine and mounted in 1% low melting agarose (Biozym, 850080) in 1x E3 medium in a glass-bottomed dish (Cellvis—D60-30-1.5-N), kept in E3 medium with 612 μM tricaine, and imaged at an upright Zeiss LSM880 system with a W-Plan Apochromat 20x/1.0 DIC objective and a 2-photon laser (Coherent Vision II) at the wavelength of 960 nm and 8.19 μs pixel dwell time. *Z*-stacks of two tiles with 10% overlap were recorded with a *z*-step of 1.23 μm. The histograms were adjusted with either ZEN Black or Adobe Photoshop. Sagittal views of dorsal *z*-stacks were created using the Reslice function in FIJI. The video was created using FIJI and Shotcut (www.shotcut.org).

### 2.7. Whole mount hybridization chain reaction RNA-FISH

Whole mount HCR RNA-FISH stainings were performed as described in the manufacturer's protocol (Molecular Instruments; https://files.molecularinstruments.com/MI-Protocol-HCRv3-Zebrafish-Rev7.pdf). The ssDNA oligos used in the corresponding oligo mixes for the mRNAs *dbh, ddc, slc6a3, slc18a2*, and *th* are listed in Supplementary Table S1. The following hairpin pairs (h1 and h2) were used: B4 Alexa Fluor^®^ 546; B2 Alexa Fluor^®^ 647; and B3 Alexa Fluor^®^ 647 (all purchased at Molecular Instruments). After the staining procedure, the larvae were stored in 80% glycerol in PBST at 4°C. The whole-mount HCR stainings were recorded using either an upright or inverted Zeiss LSM 880 system with the following lens: LD-LCI Plan Apochromat 25x/0.8 DIC. The histograms were adjusted linearly with ZEN Black.

### 2.8. Assembly of composite figures

All figures were assembled using Adobe Photoshop versions from CS4 to 24.1.1. Individual image planes, stack or sub-stack *Z*-projections, or orthogonal projections were exported as TIFF 8-bit images from ZEN or FIJI. Visualization of somata and projections from confocal image stacks was difficult due to the very different pixel intensity values, caused, first, by cytoplasmic GFP being predominantly localized to somata but not projections, and second, by fluorescence efficiency loss in deep ventral brain regions in the dorsal view recorded stacks. To better visualize both somata and projections across deep image stacks, we adjusted for individual image panels the whole image using non-linear gamma adjustments. The following steps were applied in Photoshop: whole individual images were selected and Photoshop Image Adjustment Levels menu applied, first, making minor linear adjustments to fill the 8-bit histogram without cutting information, and second, making non-linear adjustment of the mid-tone slider to make image content in the darker pixel range better visible. Therefore, signals in the image panels represent the anatomical location of fluorescence but do not linearly represent fluorescence intensities.

## 3. Results

### 3.1. Generation of a Q-system driver line in catecholaminergic neurons

**Figure 1:**
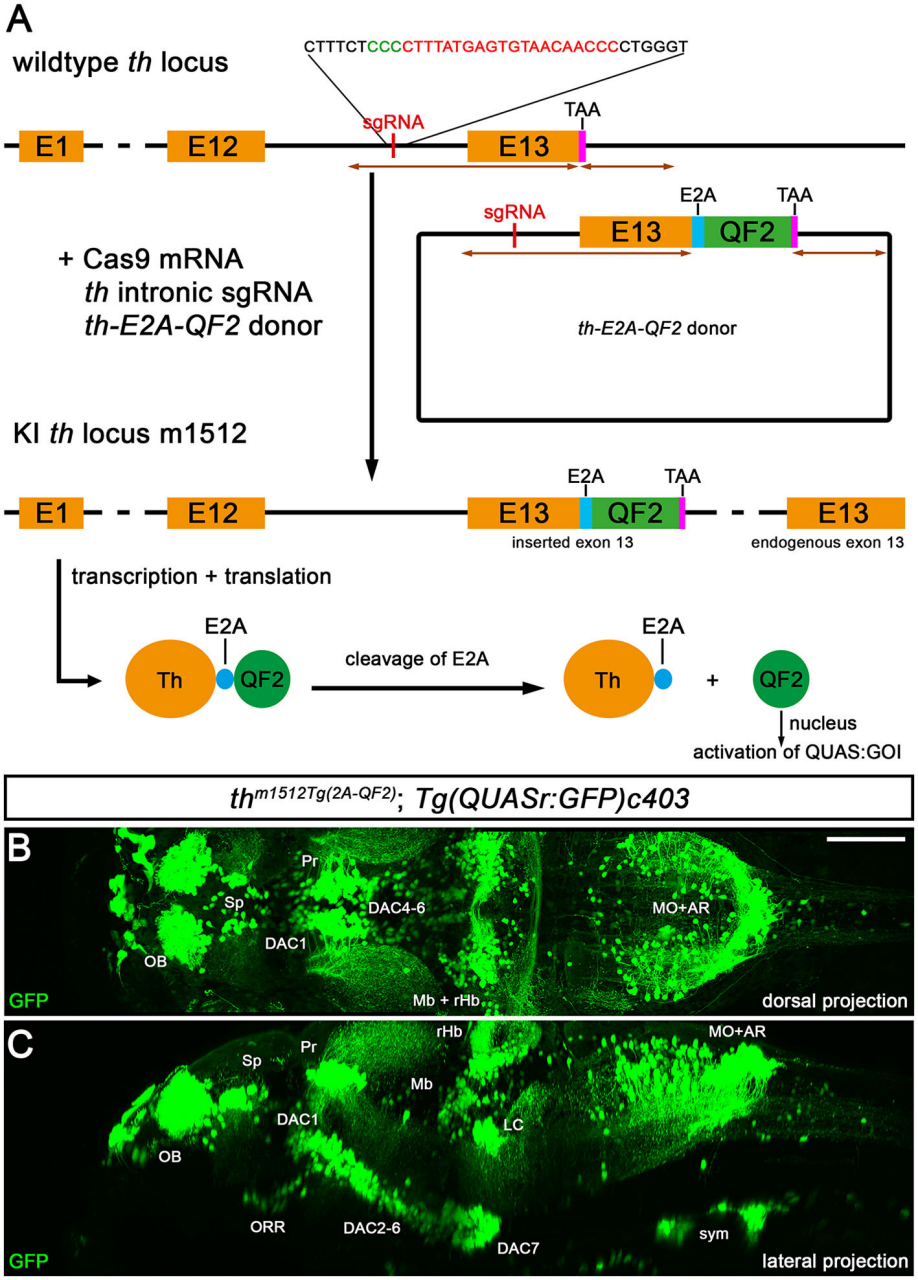
Knock-in of QF2 into the *th* locus. **(A)** Schematic of the knock-in strategy used for the generation of *th* ^*Tg*(2*A*−*QF*2)^, which is based on a previously published strategy (Li et al., 2015). Successful knock-in leads to an in-frame fusion of Th with E2A-QF2, which is cleaved after translation, releasing QF2. QF2 translocates to the nucleus and activates a gene of interest (GOI) downstream of a QUAS regulatory element. **(B)** Maximum intensity projection of a 2-photon confocal image stack of an *in vivo* recorded *th*^*m1512Tg*(2*A*−*QF*2)^*; Tg(QUASr:GFP)c403* larva at 5 dpf in a dorsal view. **(C)** Maximum intensity projection of the embryo shown in **(B)** in a sagittal resliced view. Anterior is to the left. Scale bar: 100 μm. Due to very strong intensity differences in cytoplasmic GFP fluorescence of somata vs. axons, and in order to visualize both somata and projections, we needed to record under conditions that oversaturate pixels in many somata. AR, area postrema; DAC, dopaminergic cluster; LC, locus coeruleus; Mb, midbrain; MHB, midbrain–hindbrain boundary; MO, medulla oblongata; OB, olfactory bulb; ORR, optic recess region; Pr, pretectum; rHb, rostral hindbrain; Sp, subpallium; sym, sympathetic neurons.

To drive the expression of genes of interest in catecholaminergic neurons in zebrafish, we utilized CRISPR/Cas9-mediated genome editing to insert the Q-system transcription factor QF2 (Potter et al., 2010; Riabinina et al., 2015) into the *th* locus. The Q-system in zebrafish has been reported to be less prone to transcriptional silencing in comparison with the widely used Gal4/UAS system (Subedi et al., 2014; Ghosh and Halpern, 2016; Burgess et al., 2020). We targeted the *tyrosine hydroxylase* (*th*) gene using a previously described knock-in strategy (Li et al., 2015) to generate an open reading frame fusion of Th with E2A-QF2. Injection of Cas9 mRNA, a sgRNA targeting the last intron of *th*, and a donor plasmid in single-cell stage embryos resulted in a successful knock-in of QF2 (Figure 1). The donor plasmid contains parts of the last intron of *th* such as the sgRNA target sequence, exon 13 of *th* fused with an E2A-QF2 encoding sequence, and 668 bp of genomic sequences downstream of the *th* gene (Figure 1A). After injection and rearing of G0 and F1 generation, we identified three independent *th*^*Tg*(2*A*−*QF*2)^F2 alleles: *th* ^*m1512Tg*^; *th* ^*m1513Tg*^; *th* ^*m1514Tg*^. All three independent alleles show similar expression of GFP when crossed with *Tg(QUASr:GFP)c403* (shown for *th* ^*m1513Tg*^ in Supplementary Figure S1). We chose *th* ^*m1512Tg*^ for further analysis of our knock-in transgenic line and will use *th* ^*m1512Tg*(2*A*−*QF*2)^ as a synonym for this allele throughout this study.

Using *in vivo* 2-photon microscopy, we generated an overview of GFP expression in *th* ^*m1512Tg*(2*A*−*QF*2)^*, Tg(QUASr:GFP)c403* transgenic larvae at 5 dpf (Figures 1B, C), which revealed GFP in all previously described larval DA groups in the forebrain: in the olfactory bulb, subpallium, pretectum, optic recess region (ORR; previously called the preoptic area in teleosts), and in the groups numbered 1–7 in the ventral thalamus, posterior tuberculum, and hypothalamus. Furthermore, all NA groups in the hindbrain (medulla oblongata, area postrema, locus coeruleus), and NA sympathetic neurons in the peripheral nervous system, express GFP (for CA group nomenclature, Rink and Wullimann, 2002; Filippi et al., 2014). Furthermore, we observed prominent GFP expression in the NA cells of the carotid body (data not shown). In addition, we detected GFP+ cells in the midbrain and the rostral hindbrain (rHb) close to the midbrain–hindbrain boundary (MHB), which do not belong to previously identified Th+ populations. These GFP+ cells are not a population that would only transiently express GFP, as we also observed these cells at more advanced larval stages, such as 15 dpf (Supplementary Video S1) and 30 dpf (Supplementary Figure S4). Moreover, the number of rHb GFP+ cells close to the MHB increases during development into juvenile stages (Figures 1B, C; Supplementary Video S1). The rHb GFP+ cells adjacent to the MHB appear to send out axons to the contralateral side and project to the dorsal cerebellum (Figures 1B, C; Supplementary Video S1). For technical reasons, we did not investigate *th*^*m1512Tg*(2*A*−*QF*2)^ expression in the adult zebrafish brain; however, we note that analyses of adult stages will be important to better understand the significant changes in the catecholaminergic systems from larval to juvenile and adult stages. The QF2-driver line in combination with photoactivation of Cre^ERT^ (Sinha et al., 2010) and QUAS:floxed fluorescent transgenic responders, when available, may provide an opportunity to address the anatomical correlation and differences between larval and adult catecholaminergic groups.

**Figure 2:**
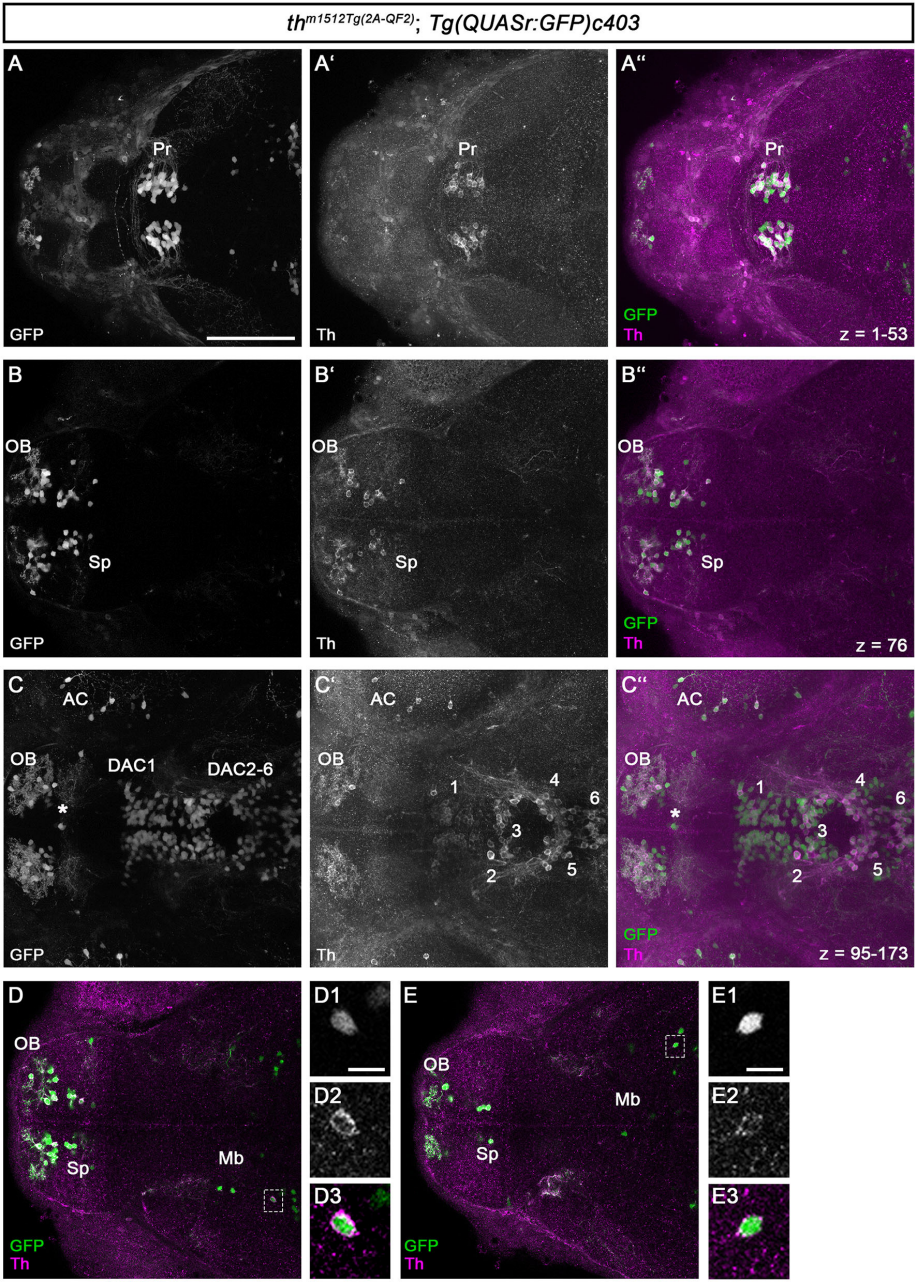
QF2-driven GFP expression highly coincides with endogenous Th. **(A–C”)** Immunofluorescence staining for GFP (green) and Th (magenta) of a *th* ^*m1512Tg*(2*A*−*QF*2)^; *Tg(QUASr:GFP)c403* embryo at 96 hpf. Dorsal views of z-projections **(A–A”, C–C”)** or single focal planes **(B–B”)**. Anterior is to the left. **(A–C”)** The expression of GFP and Th coincides in the pretectum **(A–A”)**, telencephalon **(B–B”)**, prethalamus, and hypothalamus **(C–C”)**. The total z-stack volume of 198 focal planes and *z*-step of 1 μm with focal plane 1 being the most dorsal and focal plane 198 the most ventral. **(A)** *z*-projection from focal plane 1–53. **(B)** Confocal plane 76 **(C)** *z*-projection from focal plane 95–173. The asterisk in **(C–C”)** marks a GFP but not Th-immunoreactive cell at the telencephalic midline ventricular wall. **(D, E)** Immunofluorescence staining for GFP (green) and Th (magenta) of a *th*^*m1512Tg*(2*A*−*QF*2)^; *Tg(QUASr:GFP)c403* embryo at 96 hpf with focus on GFP+ cells located in the midbrain (dashed boxes). Dorsal views of single focal planes. **(D1–D3)** Magnification of the marked cell in **(D)**. **(E1–E3)** Magnification of the marked cell in **(E)**. Scale bars: **(A–C”, D, E)** 100 μm; **(D1, E1)** 10 μm. For better representation of low and high signal intensities, non-linear adjustments were made to whole image panels (see Section 2.8). AC, amacrine cells; DAC1-6, dopaminergic cluster 1-6; Mb, midbrain; OB, olfactory bulb; Pr, pretectum; Sp, subpallium.

To determine whether all GFP+ cells also express Th, we performed whole-mount immunofluorescence for GFP and Th on *th* ^*m1512Tg*(2*A*−*QF*2)^*; Tg(QUASr: GFP)c403* heterozygous embryos at 96 hpf (Figure 2). An analysis of the DA neurons in the forebrain showed a strong coincidence of GFP and Th expression. Very few cells expressed GFP but were not Th immunoreactive (Figures 2C–C”: a cell at the telencephalic midline ventricular wall marked by an asterisk), which may represent cells that only transiently express Th but have retained GFP because the binary QF2 expression system enhances both the level and time of GFP expression or may represent ectopic transgene expression. Because we did not consistently observe such GFP-positive Th-negative cells in different larvae, we favor the idea that these cells transiently express QF2 from the *th* locus. Transient TH-expressing populations have also been documented in the developing human brain (Puelles and Verney, 1998).

All DA neurons in the pretectum express both GFP and Th (Figures 2A–A”). The DA neurons in the olfactory bulb and the subpallium show both GFP and Th signals (Figures 2B–B”). This is also the case for the DA neurons in the optic recess region (Supplementary Figure S2). For the DA groups initially numbered 1–7 (Rink and Wullimann, 2002), we will refer to them as dopamine cluster (DAC) 1–7 to indicate that we are focusing on the DA cluster. We use the DAC1–DAC7 nomenclature because the exact correlation of the early larval DA clusters with the anatomically defined clusters in the adult brain is still not fully understood (Kaslin and Panula, 2001; Rink and Wullimann, 2002; Yamamoto and Vernier, 2011). The DA clusters in the prethalamus (DAC1), in the posterior tuberculum and hypothalamus (DAC2-6; Figures 2C–C”), and the posterior recess region (DAC7; Supplementary Figure S2) coexpress GFP and Th. We found that Th is expressed at much lower levels in SP, DAC1, and DAC7 DA neurons, while the amplification by QF2 in the transgene drives higher levels of GFP expression, generating bright GFP+ signals. We also analyzed the previously uncharacterized GFP+ cells in the midbrain for Th coexpression (Figures 2D, E). We find that these Mb cells also express Th, albeit at low levels compared with the catecholaminergic groups. The magnification of two exemplary GFP+ cells in the midbrain (Figures 2, D1–D3, E1–E3) demonstrates that the brightly stained GFP+ somata also have a clear cytoplasmic halo of Th immunoreactivity. To determine whether these cells are located in the midbrain, we first performed immunofluorescence in combination with nuclear staining using TOTO-3 (Supplementary Figure S3). These data show that the Th+ and GFP+ cells are indeed located in the midbrain and not in the diencephalon (Supplementary Figures S3A”', B”', C”'). These cells most likely reside in the tegmental part of the midbrain, as their location appears ventral to the optic tectum in sagittal optical sections (Supplementary Figures S3C–C”').

We investigated whether midbrain *th* mRNA expression had been previously detected in high-quality HCR mRNA *in situ* expression data and identified an entry in the “mapzebrain” gene expression atlas (Shainer et al., 2023), showing *th* HCR data in 6 dpf larvae. The data (https://api.mapzebrain.org/media/Lines/th/average_data/T_AVG_th.zip) also revealed *th*-expressing cells in a similar location of the midbrain. To determine whether the GFP+ cells in the midbrain still express Th at juvenile stages, we performed anti-Th and anti-GFP immunofluorescence on dissected brains of 30 dpf zebrafish (Supplementary Figure S4). As a control, we recorded GFP and Th immunofluorescence in the telencephalon and pretectum (Supplementary Figures S4A, B). We found a strong coincidence of GFP and Th expression in both brain regions, with very few cells that are only Th+ but not GFP+ in the subpallium (Supplementary Figure S4A). These neurons could potentially be recently added new dopaminergic neurons, which may start to express GFP only after a short delay due to the time required for the QF2 expression and its re-entry into the nucleus to initiate GFP expression. The analysis of GFP+ cells in the midbrain revealed that these cells express low levels of Th at 30 dpf, similar to 5 dpf larval stages, (Supplementary Figure S4C and magnified area in C1–C3).

### 3.2. Th-expressing cells in the midbrain do not express other monoaminergic and catecholaminergic marker genes

**Figure 3:**
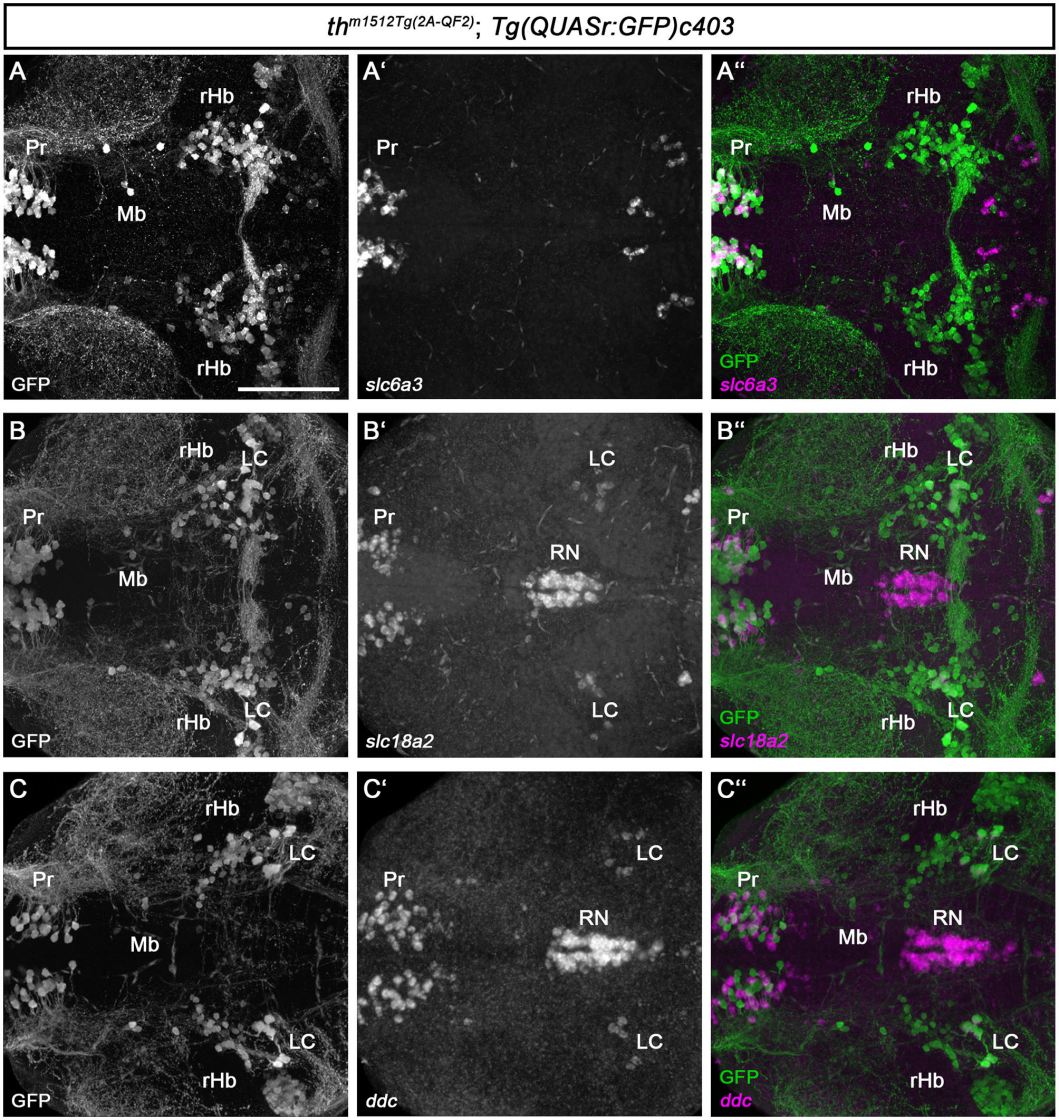
Midbrain and rHb GFP+ cells do not express other monoaminergic/catecholaminergic markers. **(A–C”)** Whole brain fluorescent *in situ* hybridization for the indicated mRNAs (magenta) and immunofluorescence staining for GFP (green) in *th*^*m1512Tg*(2*A*−*QF*2)^; *Tg(QUASr:GFP)c403* larval brains at 120 hpf. Dorsal views of z-projections. Anterior is to the left. **(A–A”)** Expression of the dopaminergic marker *slc6a3* in comparison with GFP expression in the pretectum, midbrain, and hindbrain. **(B–B”)** Expression of the monoaminergic marker *slc18a2* in comparison with GFP expression in the pretectum, midbrain, and hindbrain. **(C–C”)** Expression of the catecholaminergic marker *ddc* in comparison with GFP expression in the pretectum, midbrain, and hindbrain. Scale bar: 100 μm. For better representation of low and high signal intensities, non-linear adjustments were made to whole image panels (see Section 2.8). LC, locus coeruleus; MHB, midbrain–hindbrain boundary; Mb, midbrain; Pr, pretectum; rHb, rostral hindbrain; RN, raphe nucleus.

Catecholaminergic and serotonergic neurons share common enzymes and transporters for transmitter synthesis and processing (Flames and Hobert, 2011). To characterize the newly identified Mb and rHb Th-expressing cells, we analyzed the expression of the dopamine transporter *slc6a3*/*dat*, which is important for the reuptake of dopamine released into the synaptic cleft (Figures 3A–A”). *slc6a3* is often expressed in DA neurons, and even though some mismatches between the expression of Th and *slc6a3* have been reported (Holzschuh et al., 2001; Yamamoto et al., 2011), their combined expression may be a good indicator of a functional DA neuron. Whole brain FISH revealed no coexpression of GFP and *slc6a3* in the midbrain of *th* ^*m1512Tg*(2*A*−*QF*2)^*; Tg(QUASr:GFP)c403* brains at 5 dpf (Figure 3A”, cell marked with Mb). The enzyme Dopa decarboxylase, Ddc, and the monoamine transporter, Slc18a2/Vmat2, are required for the synthesis of dopamine and serotonin and their uptake into vesicles, respectively (Flames and Hobert, 2011). Therefore, we analyzed their expression using whole brain FISH, in *th* ^*m1512Tg*(2*A*−*QF*2)^*; Tg(QUASr:GFP)c403* brains at 5 dpf. GFP-expressing cells in the midbrain and close to the MHB do not express the monoaminergic marker *slc18a2*, in contrast to other known monoaminergic neuron groups, e.g., in the pretectum or the LC (Figures 3B–B”). Similarly, we found no coexpression of *ddc* with GFP in the Mb and rHb clusters (Figures 3C–C”).

**Figure 4:**
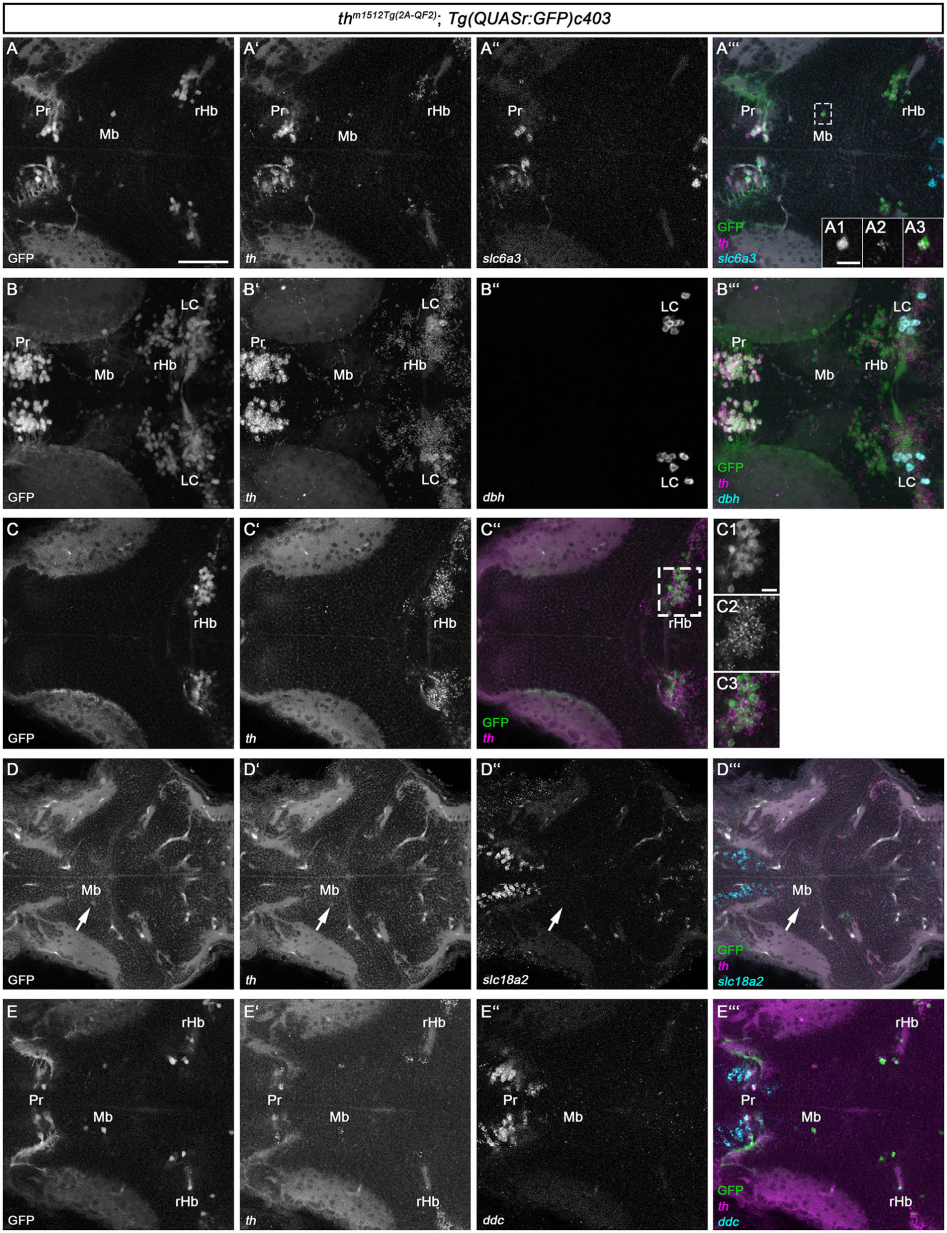
HCR RNA-FISH does not reveal coexpression of catecholaminergic markers except *th* in GFP+ cells in the midbrain and rostral hindbrain. **(A–E”')** Whole mount HCR RNA-FISH for *th* (magenta), *slc6a3* (cyan), *dbh* (cyan), *slc18a2* (cyan), and *ddc* (cyan) in comparison with GFP in *th*^*m1512Tg*(2*A*−*QF*2)^; *Tg(QUASr:GFP)c403* larvae at 120 hpf. Dorsal views of z-projections **(B–B”')** or single planes **(A–A”'**, **A1–A3**, **C–C”, D–E”')**. Anterior is to the left. **(A–A”')** Expression of *th* and *slc6a3* in comparison with GFP in the pretectum, midbrain, and hindbrain. The dashed box indicates GFP+ cells in the midbrain. **(A1–A3)** Magnification of the cell marked by the dashed box showing GFP in comparison with *th* expression. **(B–B”')** Expression of *th* and *dbh* in comparison with GFP in the pretectum, midbrain, and rostral hindbrain. **(C–C”)** Comparison of GFP with *th* expression in the rostral hindbrain close to the midbrain–hindbrain boundary, more dorsally than **(A–A”')**. **(C1–C3)** Magnification of rHb cells marked by a dashed box in **(C”)**. **(D–D”')** Expression of *th* and *slc18a2* in comparison with GFP expression in the midbrain. **(E–E”')** Comparison of GFP expression with *th* and *ddc* in the pretectum, midbrain, and rostral hindbrain close to the midbrain–hindbrain boundary. Scale bars: **(A)** 50 μm; **(A1)** 10 μm; **(C1)** 10 μm. For better representation of low and high signal intensities, non-linear adjustments were made to whole image panels (see Section 2.8). LC, locus coeruleus; Mb, midbrain; MHB, midbrain–hindbrain boundary; Pr, pretectum; rHb, rostral hindbrain.

The whole brain FISH technique is suitable for detecting medium to high levels of expression but often fails to detect low-level gene expression. As the Mb and rHb GFP+ cells express low levels of Th protein, and Th protein in some cells is undetectable, we made use of the recently established HCR RNA-FISH 3.0 technique (Choi et al., 2018), which enables the detection of low-level transcripts. We designed HCR probes for *th, dbh, ddc, slc6a3*, and *slc18a2* (Supplementary Table S1) and analyzed *th* ^*m1512Tg*(2*A*−*QF*2)^*; Tg(QUASr:GFP)c403* larvae at 5 dpf (Figure 4). Our HCR RNA-FISH analysis confirmed that GFP+ cells in the midbrain (Figures 4A, A', A”' and magnified cell in A1-A3) and rostral hindbrain (Figures 4C–C” and magnified cells in C1-C3) express low levels of *th* mRNA. To exclude that sequences of the QF2 transgene, including the plasmid backbone, might cause ectopic expression of *th* in these cells, we performed *th* HCR RNA-FISH on wild-type 96 hpf zebrafish (Supplementary Figure S5). As expected, we observed high expression of *th* in the A11-type neurons in the posterior tuberculum and hypothalamus (Supplementary Figure S5A). In addition, we found cells expressing low levels of *th* in the midbrain (Supplementary Figures S5B, B') and in the rostral hindbrain close to the MHB (Supplementary Figures S5C, C'). These findings confirm that Mb and rHb *th*+ are indeed endogenously expressing *th* in wild-type zebrafish. However, these GFP+ and *th*+ cells do not express the DA marker *slc6a3*, as we detected no transcripts in these cells (Figures 4A”, A”'). NA neurons express, in contrast to DA neurons, the enzyme dopamine beta-hydroxylase, Dbh, which catalyzes the conversion of dopamine to noradrenaline. Therefore, *dbh* expression is used to identify NA neurons in zebrafish (Guo et al., 1999). Expression analysis of *dbh* through HCR RNA-FISH revealed that the Mb and rHb GFP+ cells do not express *dbh* (Figures 4B–B”'). Thus, we do not consider these cells to be NA neurons. In addition, we performed HCR RNA-FISH for *ddc* and *slc18a2*, but did not observe any coexpression of these markers in Mb or rHb GFP+ cells at 5 dpf (Figures 4D–E”'). Catecholaminergic neurons synthesize, in addition to dopamine, other neurotransmitters like glutamate or GABA (Filippi et al., 2014). We found the neurons in the midbrain to be GABAergic, as immunofluorescence at 96 hpf revealed that they express the enzyme glutamate decarboxylase, Gad1b, or Gad2 (Supplementary Figure S6). In conclusion, the newly identified Th-expressing cells in the midbrain and rostral hindbrain lack the expression of key enzymes of dopamine and noradrenaline synthesis but may be GABAergic.

### 3.3. Th-expressing cells located in the tegmentum and rostral hindbrain are axon-projecting

**Figure 5:**
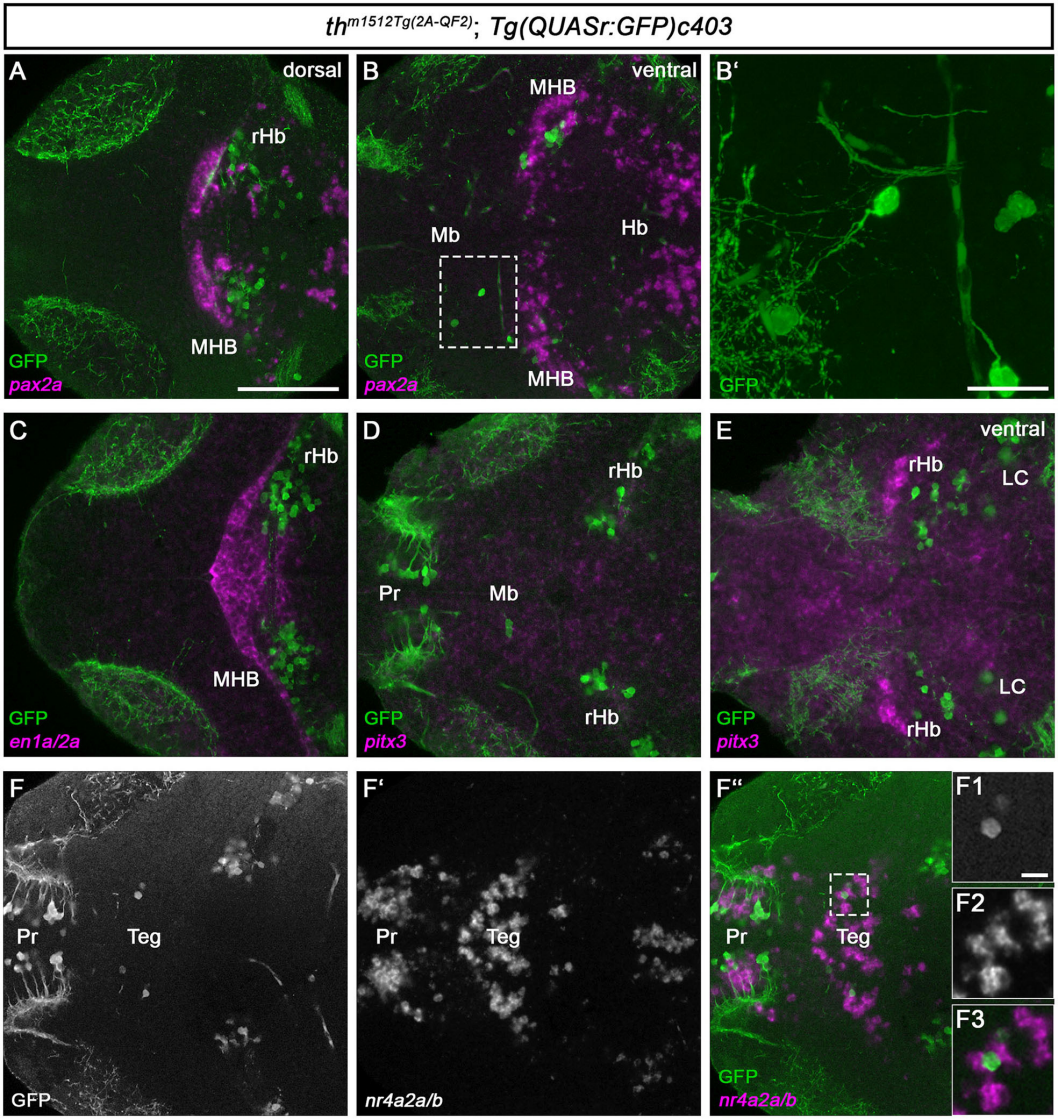
Th/*th*+ neurons reside in the tegmental midbrain and the rostral hindbrain. **(A–F”**, **F1–F3)** Whole-brain fluorescent *in situ* hybridization and immunofluorescence staining for the indicated mRNAs (magenta) and GFP (green) in *th*^*m1512Tg*(2*A*−*QF*2)^; *Tg(QUASr:GFP)c403* larval brains at 120 hpf. Dorsal views of z-projections **(B')** or single planes **(A, B, C–F”**, **F1-F3)**. Anterior is to the left. **(A, B)** Expression of the midbrain–hindbrain boundary marker *pax2a* in comparison with GFP in a dorsal view **(A)** and a more ventral view **(B)**. The dashed box indicates GFP+ cells in the midbrain. **(B')** z-projection (z-stack size: 31 μm) and magnification of the cells marked by a dashed box in **(B)**. **(C)** Expression of the midbrain–hindbrain boundary marker *en1a/en2a* in comparison with GFP. **(D, E)** Expression of the mammalian midbrain dopaminergic marker *pitx3* in comparison with GFP, focusing on GFP+ cells in the midbrain **(D)** and a more ventral view **(E)**. **(F–F”)** Expression of the mammalian mDA and tegmental marker *nr4a2a/nr4a2b* in comparison with GFP. The dashed box in **(F”)** indicates GFP+ cells in the midbrain. **(F1–F3)** Magnification of the cells marked by a dashed box in **(F”)**. Scale bars: [**(A)**, also for **(B, C–F”)**] 100 μm; **(B')** 20 μm; **(F1–F3)** 10 μm. For better representation of low and high signal intensities, non-linear adjustments were made to whole image panels (see Section 2.8). LC, locus coeruleus; Mb, midbrain; MHB, midbrain–hindbrain boundary; Pr, pretectum; rHb, rostral hindbrain; Teg, tegmentum.

DA neurons in the mammalian midbrain are characterized by the expression of several transcription factors that define their regional identity and differentiation (Filippi et al., 2007; Arenas et al., 2015; Blaess and Ang, 2015). To determine whether the zebrafish Mb Th-expressing cells might share the expression of homologs of these factors, we analyzed the expression of *en1a/2a, pitx3*, and *nr4a2a/b* (Figure 5). We also analyzed the expression of *pax2a* and *en1a/2a* as anatomical markers of the MHB to clarify the exact location of the rHb GFP+ cells. The *pax2a* expression domain appears rostrally adjacent and in part intermingled with the rHb GFP+ cells at 5 dpf, indicating that the rHb GFP+ cells indeed are located in the rostral hindbrain (Figures 5A, B). Consistent with this finding, *en1a/2a* expression is rostrally adjacent to rHb Th+ cells (Figure 5C). Neither the Mb nor the rHb GFP+ cells express *pitx3* (Figures 5D, E). In contrast, the Mb GFP+ cells express *nr4a2a/b* or are at least located in a *nr4a2a/b*-positive region of the midbrain (Figures 5F–F” and magnified cells in F1-F3). To clarify whether the Mb GFP+ cells are *bona fide* neurons, we imaged them at higher magnification (Figure 5B and magnified cells in Figure 5B'). These two GFP+ cells do indeed send out axons and are therefore likely to be neurons and not immature progenitor cells (Figure 5B'). Unfortunately, because the Mb GFP+ cells are very rare and express low levels of GFP, a mapping of potential projection targets using mosaic analysis (Tay et al., 2011) is not feasible. In conclusion, we show that *th* ^*m1512Tg*(2*A*−*QF*2)^*; Tg(QUASr:GFP)c403* larvae and juvenile fish develop previously unidentified GFP-expressing GABAergic neurons in the midbrain and GFP-expressing neurons in the hindbrain, both of which express Th/*th*, but no other catecholaminergic or mammalian midbrain DA marker genes, except *nr4a2a/b*.

## 4. Discussion

In this study, we generated a new transgenic line using a previously described CRISPR/Cas9-mediated knock-in protocol (Li et al., 2015). Our knock-in line drives the expression of the QF2 transcription factor in the CA system. We confirmed the faithful expression of the QUAS fluorescent reporter using coexpression analysis with *th* at the mRNA level and Th at the protein level. Previously, *th* and *dat/slc6a3* promoter constructs, engineered BACs, or knock-in have been used to drive expression in CA or DA neurons (Fujimoto et al., 2011; Tay et al., 2011; Fernandes et al., 2012; Godoy et al., 2015; Li et al., 2015; Shang et al., 2015; Haehnel-Taguchi et al., 2018; Gu et al., 2021; Ilin et al., 2021). While these genetic tools have provided important insights into zebrafish CA biology, most transgenic systems have not achieved homogeneous reporter expression in all DA or NA neuronal groups and may also show ectopic expression. To avoid the variable expression levels from the endogenous promoters in different anatomical groups, and to drive high expression levels, the Gal4 system was used; however, Gal4 tends to drive mosaic expression in zebrafish due to transgene inactivation (Akitake et al., 2011). To avoid mosaicism and to drive homogeneous high expression in all CA neurons, we used the QF2:QUAS binary expression system (Subedi et al., 2014; Ghosh and Halpern, 2016). Indeed, we find that our *th* ^*m1512Tg*(2*A*−*QF*2)^ transgene drives QUAS responder expression at similar expression levels in all CA groups, largely avoiding responder expression mosaicism. One limitation remains: The existence of two *tyrosine hydroxylase* genes in teleosts (Candy and Collet, 2005; Chen et al., 2009; Filippi et al., 2010) means that *th* ^*m1512Tg*(2*A*−*QF*2)^ responders are not expressed in *th2*-only expressing CA neurons, which are mostly located in the posterior and lateral recesses in the hypothalamus, the paraventricular organ, and the optic recess region (Chen et al., 2009; Filippi et al., 2010; Yamamoto et al., 2010). The new genetic tool enables experiments that rely on homogenous expression levels of proteins (e.g., GCaMPs neuronal activity reporters, or optogenetic tools) in all CA neurons, and thereby facilitates analyses and may foster new research directions.

The bright labeling by QUAS reporters responding to the *th* ^*m1512Tg*(2*A*−*QF*2)^ QF2 driver also allowed us to identify two *th*-expressing neuronal populations in the midbrain tegmentum and the rostral hindbrain, close to the MHB. The Th-expressing cells in the zebrafish midbrain and rostral hindbrain do not express other catecholaminergic markers such as *slc6a3*/*dat, slc18a2*/*vmat2, ddc*, or the NA-specific marker *dbh*. Therefore, we do not consider these cells to be *bona fide* DA or NA neurons. However, also other previously characterized DA neuronal populations do not express all aforementioned marker genes (Fougere et al., 2021). Some propose that midbrain DA neurons are better identified by the expression of the dopamine transporter *Slc6a3* rather than *Th*, as *Th* mRNA-expressing neurons lacking *Slc6a3* expression are also found in the mammalian brain (Lammel et al., 2015; Poulin et al., 2018; Tiklova et al., 2019). In addition, single-cell transcriptomics of the human and mouse ventral midbrain identified an immature DA neuron population that already expresses *Th* but lacks *Slc18a2* and *Slc6a3* expression (La Manno et al., 2016). Hence, the Th-expressing cells in the zebrafish midbrain may be late progenitor/early mature neurons, which will later start the expression of other catecholaminergic markers. DA neurons in both mammalian and zebrafish brains produce either glutamate or GABA as secondary neurotransmitters (Kawano et al., 2006; Descarries et al., 2008; Chuhma et al., 2009; Filippi et al., 2014). Midbrain DA neurons mostly produce glutamate (Descarries et al., 2008; Chuhma et al., 2009), but recent single-cell transcriptomics of midbrain DA neurons also revealed a GABAergic subtype in the VTA (Saunders et al., 2018; Tiklova et al., 2019). We found the *th*/Th-expressing cells in the midbrain also to be GABAergic based on the coexpression of Gad1b/2.

Midbrain DA neurons are not only characterized by the expression of catecholaminergic markers but also by the expression of anatomical region- or lineage-specific transcription factors that start to be expressed during development and are often maintained in mature DA neurons (Arenas et al., 2015; Blaess and Ang, 2015). These include *Nr4a2, Pitx3*, and *En1/2*. Expression analysis of zebrafish homologs of these genes revealed that the Th+ neurons in the midbrain do not express *pitx3* and *en1a*/*en2a*. However, these neurons are located in a *nr4a2a*/*b* expressing area and some of them coexpress *nr4a2a*/*b*. This observation indicates that these newly identified neurons are part of the tegmentum since *nr4a2a*/*b* is exclusively expressed in the tegmental part of the midbrain, but not in the optic tectum (Filippi et al., 2007; Blin et al., 2008). The *th*-expressing cells close to the MHB are located posterior to *pax2a* expression (Krauss et al., 1991b) and are therefore considered to be part of the rostral hindbrain. Previously, a WISH signal for *th* mRNA was detected diffusely in the cerebellum of juvenile zebrafish, without clearly identifiable somata that would be a source of *th* mRNA (Figure 1A in Filippi et al., 2010). It has been hypothesized that these *th* transcripts may stem through axonal transport from NA neurons of the locus coeruleus innervating the cerebellum (Filippi et al., 2010; Tay et al., 2011). However, the *th* transcripts previously identified in the cerebellum may also stem from our newly identified rHb *th*+ neurons, as they show extensive innervation of the cerebellum already at larval stages. Whether Tyrosine hydroxylase potentially translated from this source in the cerebellum has any physiological function remains unclear.

The rHb GFP+ neurons express *th* mRNA, as revealed by the HCR RNA-FISH, but do not appear positive for Th protein. Similarly, in the mammalian midbrain and other brain regions, *Th* mRNA is more broadly detected than the translated TH protein (Lammel et al., 2015; Yamaguchi et al., 2015). Additionally, it has been reported that *Th* transcripts synthesized in short-term response to stimulation bind weakly to polysomes and do not undergo translation (Xu et al., 2007). The expression of *Th* mRNA and TH protein is tightly regulated at multiple levels, and the neurons we identified to be located in the rostral hindbrain may not translate Th or may only start translating *th* mRNA to Th protein upon long-term stimulation. TH-expressing neurons have also been identified in the rat and human cerebellum and have been termed A4 neurons (Dahlstrom and Fuxe, 1964; Puelles and Verney, 1998). The *th*-expressing rostral hindbrain neurons may be homologous to these mammalian A4 neurons.

Midbrain DA neurons have not been described in zebrafish (Rink and Wullimann, 2002). In contrast, midbrain DA cells have been identified in the tegmental area of cartilaginous fish (Carrera et al., 2005, 2012). Recently, several studies have reported Th-expressing neurons in different actinopterygian species in similar brain regions compared with the neurons we identified in the tegmental part of the midbrain (Lopez et al., 2019; Lozano et al., 2019; Borgonovo et al., 2021). This suggests that our newly identified Th-expressing neurons are not an exclusive feature of zebrafish but may be evolutionary remnants of the midbrain DA neurons found in cartilaginous fish, which are lost or severely reduced in most teleost species.

## Data availability statement

The original contributions presented in the study are included in the article/Supplementary material, further inquiries can be directed to the corresponding author.

## Ethics statement

The animal study was reviewed and approved by Regierungspräsidium Freiburg.

## Author contributions

WD and CA designed the study and analyzed the data. CA and JH performed the experiments. CA wrote the first draft of the manuscript and prepared all figures. WD edited the manuscript, obtained funding, and supervised the project. All authors contributed to the article and approved the submitted version.
