# Supplementary material for: CRISPR/Cas9-based QF2 knock-in at the tyrosine hydroxylase (th) locus reveals novel th-expressing neuron populations in the zebrafish mid- and hindbrain
Source: Front Neuroanat. 2023 Aug 2;17:1196868. doi: 10.3389/fnana.2023.1196868 (PMC10433395; doi:10.3389/fnana.2023.1196868)
Supplement: Supplementary file 2 [file Data_Sheet_1.pdf]

Supplementary Material

CRISPR/Cas9-based QF2 knock-in at the *tyrosine hydroxylase* (*th*) locus reveals novel *th*-expressing neuron populations in the zebrafish mid- and hindbrain

Christian Altbürger, Jens Holzhauser, Wolfgang Driever*

*** Correspondence:** Wolfgang Driever: driever@biologie.uni-freiburg.de

1 Supplementary Data

Supplementary Table 1 (pages 1 – 3)

Supplementary Figures 1 to 6 (pages 4 – 11)

Supplementary Video 1 legend (page 11)

2 Supplementary Tables

Supplementary Table 1. Sequences of the DNA oligos used for HCR RNA-FISH.

*dbh*_B2	oligo sequences
HCR_Dan_dbh_B2_1	CCTCgTAAATCCTCATCAAATCAGGGTTACGTCTTGGAGCCGGAG
HCR_Dan_dbh_B2_2	CCACAGTGGCTAAAACGGTGAGGTAAAATCATCCAgTAAACCgCC
HCR_Dan_dbh_B2_3	CCTCgTAAATCCTCATCAAATGGGATAACTGATGTTCCAGGACAG
HCR_Dan_dbh_B2_4	CTTTGACCTCGAGCAAAACCTCCTGAAATCATCCAgTAAACCgCC
HCR_Dan_dbh_B2_5	CCTCgTAAATCCTCATCAAAAAAGAAGCCTTCAGTGGACTGATGG
HCR_Dan_dbh_B2_6	GGTGCTGAATGATCTTTTGAAGAGCAAATCATCCAgTAAACCgCC
HCR_Dan_dbh_B2_7	CCTCgTAAATCCTCATCAAATCGCTGGACGCCAGGCTGGAGACGG
HCR_Dan_dbh_B2_8	CGGGGTGTCTGGACGCAGGAGCAGGAAATCATCCAgTAAACCgCC
HCR_Dan_dbh_B2_9	CCTCgTAAATCCTCATCAAACAATGTGGTTCTTGGGCAGATTTGG
HCR_Dan_dbh_B2_10	CTGGAGTGATCACTGACTCATACATAAATCATCCAgTAAACCgCC
HCR_Dan_dbh_B2_11	CCTCgTAAATCCTCATCAAACCATAGGCAAACCAGCGTCAGCAGG
HCR_Dan_dbh_B2_12	GAAGAAACCTAGAAGATCCTTCTCCAAATCATCCAgTAAACCgCC
HCR_Dan_dbh_B2_13	CCTCgTAAATCCTCATCAAAGCCCAAGCTCCATGATGCCTGCGTC
HCR_Dan_dbh_B2_14	GAATGGCCATCACAGGAGTGTAGACAAATCATCCAgTAAACCgCC
HCR_Dan_dbh_B2_15	CCTCgTAAATCCTCATCAAACGTCTGTGTGCATTTGGCGGTGCAG
HCR_Dan_dbh_B2_16	GATGTGTATGCCCCCTACTGGAAGAAAATCATCCAgTAAACCgCC
HCR_Dan_dbh_B2_17	CCTCgTAAATCCTCATCAAAATGTGTGCTGAAATGTTTGTCCTCC
HCR_Dan_dbh_B2_18	CTTCTGTAAAACACGGATGATCTGGAAATCATCCAgTAAACCgCC
HCR_Dan_dbh_B2_19	CCTCgTAAATCCTCATCAAAGATGAGGCTGAAGTATTTCTGCAGG
HCR_Dan_dbh_B2_20	GCAGCTCTCGCGGCCCTGAAACCTGAAATCATCCAgTAAACCgCC

*ddc*_B2	oligo sequences
HCR_Dan_ddc_B2_1	CCTCgTAAATCCTCATCAAACACATCCTCATAGCTCTCTGGCTCC
HCR_Dan_ddc_B2_2	CATGATCACCCTCTCGATGTCTTTGAAATCATCCAgTAAACCgCC
HCR_Dan_ddc_B2_3	CCTCgTAAATCCTCATCAAATATGCATAGAAGTATGGGCTGTGCC
HCR_Dan_ddc_B2_4	GCAGGATAAGAGTGGGCAGTGGGAAAAATCATCCAgTAAACCgCC
HCR_Dan_ddc_B2_5	CCTCgTAAATCCTCATCAAATCTCTGCATTGCATATAGGGCCCAG
HCR_Dan_ddc_B2_6	ATGCAGCATCGATGTGCATCCACATAAATCATCCAgTAAACCgCC
HCR_Dan_ddc_B2_7	CCTCgTAAATCCTCATCAAACTCCGGACAGATAAAAGCACTCCCG
HCR_Dan_ddc_B2_8	CTCAATGCCGTTCAGCAAGGGTCTGAAATCATCCAgTAAACCgCC
HCR_Dan_ddc_B2_9	CCTCgTAAATCCTCATCAAAAGTGACAAGTCCTGACTCTTGGTGA
HCR_Dan_ddc_B2_10	CAGCGGGATCTGCCAGTGTCTATAAAAATCATCCAgTAAACCgCC
HCR_Dan_ddc_B2_11	CCTCgTAAATCCTCATCAAACCAGCTGACGGATGTGGCACCAGGC
HCR_Dan_ddc_B2_12	GTAGCAGTTCCTGAAGCAGCTCTGAAAATCATCCAgTAAACCgCC
HCR_Dan_ddc_B2_13	CCTCgTAAATCCTCATCAAAACACCATTATGACAATCACGGACGC
HCR_Dan_ddc_B2_14	GCCTGGATTGCTACACACTCATGGCAAATCATCCAgTAAACCgCC
HCR_Dan_ddc_B2_15	CCTCgTAAATCCTCATCAAACGCGTCTCCGAAACTCTGCGGCATC
HCR_Dan_ddc_B2_16	CCGCCACATAATCAACCATCTCCCTAAATCATCCAgTAAACCgCC
HCR_Dan_ddc_B2_17	CCTCgTAAATCCTCATCAAAAGGGTAAACCTGCCTTTTCTCAATG
HCR_Dan_ddc_B2_18	TGACCTCAGATATCCCGGTTCCACAAAATCATCCAgTAAACCgCC
HCR_Dan_ddc_B2_19	CCTCgTAAATCCTCATCAAATCCAATGCAGCCAATGGCTCCACAC
HCR_Dan_ddc_B2_20	ACAGGCAGGACTTGCAGCCCAAGAGAAATCATCCAgTAAACCgCC

*slc6a3*_B3	oligo sequences
HCR_Dan_slc6a3_B3_1	gTCCCTgCCTCTATATCTTTAAAGTCGATCTTCTTGCCCCAGGTC
HCR_Dan_slc6a3_B3_2	CACAGCGAAGCCGATGACGGACAGCTTCCACTCAACTTTAACCCg
HCR_Dan_slc6a3_B3_3	gTCCCTgCCTCTATATCTTTCTCCTCCGCCGTTCTTGTAGCACAG
HCR_Dan_slc6a3_B3_4	TGAAGAAGAGATACGGCACCAGGAATTCCACTCAACTTTAACCCg
HCR_Dan_slc6a3_B3_5	gTCCCTgCCTCTATATCTTTGTGAAGCCCACGCCTTTGAATATGG
HCR_Dan_slc6a3_B3_6	CCCACATACAGCGAGATCAGGATCATTCCACTCAACTTTAACCCg
HCR_Dan_slc6a3_B3_7	gTCCCTgCCTCTATATCTTTGTGGCGTTGAGGTCGGAGCAGTTGG
HCR_Dan_slc6a3_B3_8	GTGGTCTTGTACGTGTCGTTGAGCATTCCACTCAACTTTAACCCg
HCR_Dan_slc6a3_B3_9	gTCCCTgCCTCTATATCTTTAGCTTTCATGAACATGCAGAACACC
HCR_Dan_slc6a3_B3_10	GCGGCGCACCCAGATCATCAATACCTTCCACTCAACTTTAACCCg
HCR_Dan_slc6a3_B3_11	gTCCCTgCCTCTATATCTTTGACCACATACGGCATCGTGGCAGTG
HCR_Dan_slc6a3_B3_12	GACGCCACGCAGGAGCAGCACGGTCTTCCACTCAACTTTAACCCg
HCR_Dan_slc6a3_B3_13	gTCCCTgCCTCTATATCTTTGAAGTCCACACTCAGGTAAGCTTTA
HCR_Dan_slc6a3_B3_14	ATGTACCTGAGCATCATACAGGCGCTTCCACTCAACTTTAACCCg
HCR_Dan_slc6a3_B3_15	gTCCCTgCCTCTATATCTTTATGGAGCTGGTGATGATGGCGTCTC
HCR_Dan_slc6a3_B3_16	CCGGAGAAGAAGCTGGTCAGAGAGTTTCCACTCAACTTTAACCCg
HCR_Dan_slc6a3_B3_17	gTCCCTgCCTCTATATCTTTGGGCAGCGTCGCAATGGCTTCTGGG
HCR_Dan_slc6a3_B3_18	GAAGATGACGGCCCACACTGAAGAGTTCCACTCAACTTTAACCCg
HCR_Dan_slc6a3_B3_19	gTCCCTgCCTCTATATCTTTTGAAGAGTGTGAAGAGCTCTCGATG
HCR_Dan_slc6a3_B3_20	GCGAGATGAGGAAGGTGGACACCACTTCCACTCAACTTTAACCCg

*slc18a2*_B3	oligo sequences
HCR_Dan_slc18a2_B3_1	gTCCCTgCCTCTATATCTTTGTGAAAGGCACGCTGGATGCGGAGC
HCR_Dan_slc18a2_B3_2	GCATTGAGCTTTGCTGTCAGATCCCTTCCACTCAACTTTAACCCg
HCR_Dan_slc18a2_B3_3	gTCCCTgCCTCTATATCTTTAGGCACGACCACCGTCAACAGCATG
HCR_Dan_slc18a2_B3_4	CACCGTGTACAGGTAACTTGGGATATTCCACTCAACTTTAACCCg
HCR_Dan_slc18a2_B3_5	gTCCCTgCCTCTATATCTTTACCAGCTGTGCTCATCTGCGGGCTG
HCR_Dan_slc18a2_B3_6	CACAAAAGTAGGAGCCAGGCTCATTTTCCACTCAACTTTAACCCg
HCR_Dan_slc18a2_B3_7	gTCCCTgCCTCTATATCTTTTCTCGTTCAGAAGCTGGTCATCTGC
HCR_Dan_slc18a2_B3_8	AGGCAAACAACAGACCCACTTTCACTTCCACTCAACTTTAACCCg
HCR_Dan_slc18a2_B3_9	gTCCCTgCCTCTATATCTTTGCAGCGTGTAGCTTGATGAAAAGGC
HCR_Dan_slc18a2_B3_10	CACCTTGCAGAGATCTGGCCAAAAATTCCACTCAACTTTAACCCg
HCR_Dan_slc18a2_B3_11	gTCCCTgCCTCTATATCTTTCAACTAAAACTCCCATTGCTAGTCC
HCR_Dan_slc18a2_B3_12	CATACATGACACTGCCAAATGGAGGTTCCACTCAACTTTAACCCg
HCR_Dan_slc18a2_B3_13	gTCCCTgCCTCTATATCTTTTCCATCAAGCACTGCCAGCACTGCC
HCR_Dan_slc18a2_B3_14	AGGCTGAAGCACAAAAAGCTGCAGATTCCACTCAACTTTAACCCg
HCR_Dan_slc18a2_B3_15	gTCCCTgCCTCTATATCTTTTTCCTTTTTGACTCTCTGGTTCCAC
HCR_Dan_slc18a2_B3_16	GGTCCTTCATGAGGGTTATAAGTGATTCCACTCAACTTTAACCCg
HCR_Dan_slc18a2_B3_17	gTCCCTgCCTCTATATCTTTCCATCATCCAGATGGGCAACGCCGG
HCR_Dan_slc18a2_B3_18	GCTGCCATTTCCGGGGGCACATGGTTTCCACTCAACTTTAACCCg
HCR_Dan_slc18a2_B3_19	gTCCCTgCCTCTATATCTTTATACTGATTCCAACCAGCAGCATGC
HCR_Dan_slc18a2_B3_20	ATGTCTTTTGCAAGAGGCACGCAGATTCCACTCAACTTTAACCCg

*th*_B4	oligo sequences
HCR_Dan_th_B4_1	CCTCAACCTACCTCCAACAAAAGATGTGGAGCTGCTTGAATTCGG
HCR_Dan_th_B4_2	ACGCAGCTCTCCGGATGCTCTTTGTATTCTCACCATATTCgCTTC
HCR_Dan_th_B4_3	CCTCAACCTACCTCCAACAACCAACAAACTTCTGAGACGTAATAG
HCR_Dan_th_B4_4	GCATCCTCGATCAAACTCTGCCGCCATTCTCACCATATTCgCTTC
HCR_Dan_th_B4_5	CCTCAACCTACCTCCAACAAGCTGGGTCTGGTTTCAAGATGGTGG
HCR_Dan_th_B4_6	GTCCTCCAAGCCATCCTTTGGTTTTATTCTCACCATATTCgCTTC
HCR_Dan_th_B4_7	CCTCAACCTACCTCCAACAAAACATCCGACAGGTGCACTTCACAC
HCR_Dan_th_B4_8	TCTCTTCAAAGAGCTGACCAGCGTGATTCTCACCATATTCgCTTC
HCR_Dan_th_B4_9	CCTCAACCTACCTCCAACAAGGTGATCCTGATCCAGATCCGGATC
HCR_Dan_th_B4_10	TTCTGTAAACAGGGTCAGTAAATCCATTCTCACCATATTCgCTTC
HCR_Dan_th_B4_11	CCTCAACCTACCTCCAACAATCTGTATTTGAAGGCAATGTCTCCG
HCR_Dan_th_B4_12	GTCCACTCTAGGAATTGGTTCTCCAATTCTCACCATATTCgCTTC
HCR_Dan_th_B4_13	CCTCAACCTACCTCCAACAAAGCCGCAATGTTTCTCCAGTAACCG
HCR_Dan_th_B4_14	CCAGCTGAGGGATGTTATCTGGGCTATTCTCACCATATTCgCTTC
HCR_Dan_th_B4_15	CCTCAACCTACCTCCAACAACACTGGCCTCAACTGAAATCCTGTG
HCR_Dan_th_B4_16	AAAATCGCGTGCTGAGAGCAAACCTATTCTCACCATATTCgCTTC
HCR_Dan_th_B4_17	CCTCAACCTACCTCCAACAAAGTGAACCAGTACATTGTCGATAGC
HCR_Dan_th_B4_18	TCCCTGTTTACACAGTCCAAACTCCATTCTCACCATATTCgCTTC
HCR_Dan_th_B4_19	CCTCAACCTACCTCCAACAAGGTAAGTCTGGTCTTGGTATGGCTG
HCR_Dan_th_B4_20	AGCTTTCTGACACAAAATAGACAGGATTCTCACCATATTCgCTTC

3 Supplementary Figures

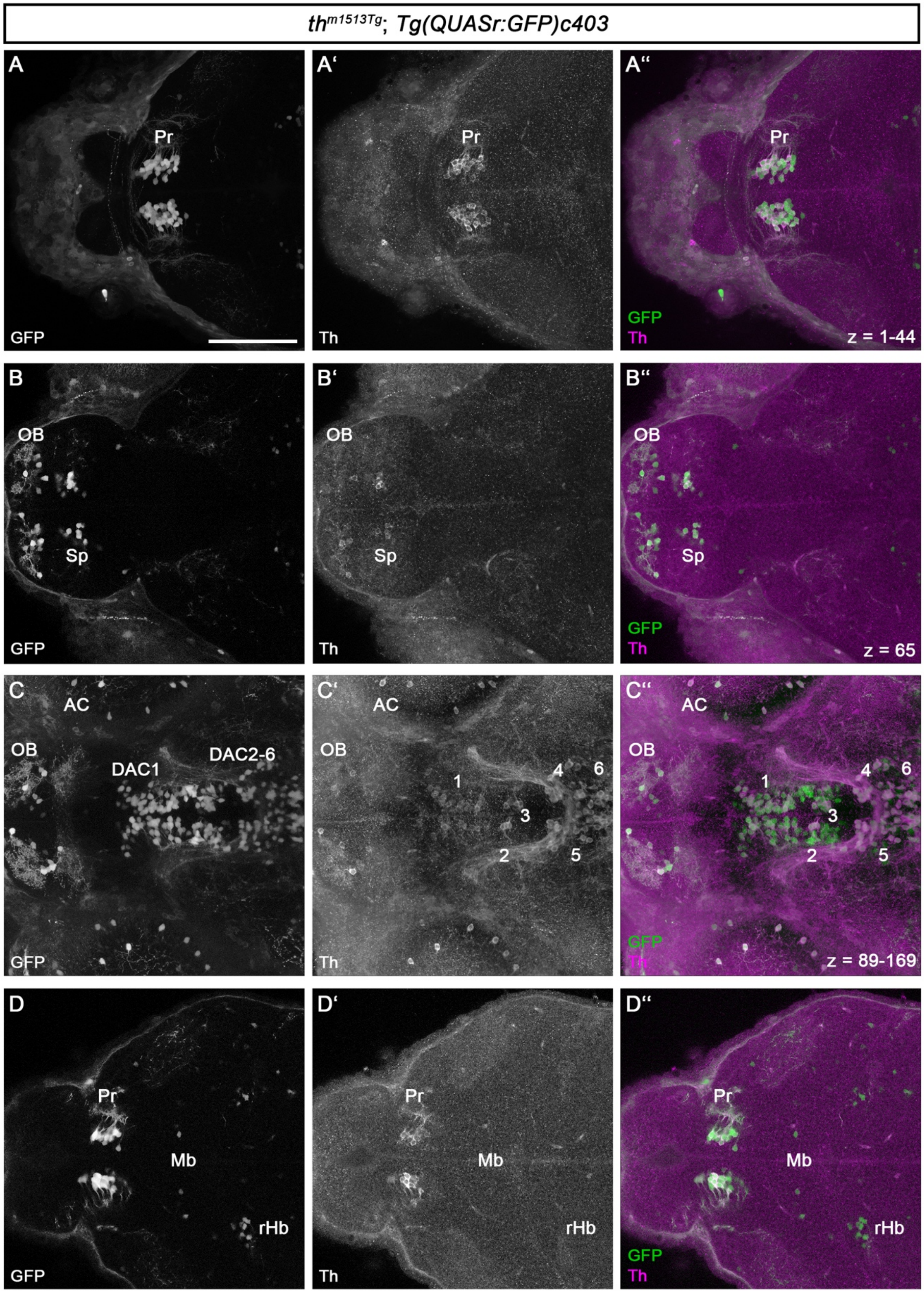

Supplementary Figure 1. ***th*** $^{m1513Tg(E2A\text{-}QF2)}$ **driven GFP expression coincides with Th expression.**

In this figure, we characterize the second independent *th* $^{m1513Tg(E2A-QF2)}$ knock-in allele to be expressed identical to the *th* $^{m1513Tg(E2A-QF2)}$ allele used in all other figures of this study. **(A-C'')** Whole-mount immunofluorescence staining for GFP (green) and Th (magenta) of a *th* ^{m1513Tg}; *Tg(QUASr:GFP)c403* embryo at 96 hpf. Dorsal views of z-projections (**A-A''**, **C-C''**) or single focal planes (B-B''). Anterior is to the left. **(A-C'')** The expression of GFP and Th overlaps in the pretectum (**A-A''**), telencephalon (**B-B''**), prethalamus, posterior tuberculum and hypothalamus (**C-C''**). Total z-stack volume of 169 focal planes and z-step of 1.0 µm with focal plane 1 being most dorsal and focal plane 169 most ventral. (**A**) z-volume from focal plane 1 to 44. (B) focal plane 65 (**C**) z-volume from focal plane 89 to 169. **(D-D'')** Immunofluorescence staining for GFP (green) and Th (magenta) of a *th* ^{m1513Tg}; *Tg(QUASr:GFP)c403* embryo at 96 hpf with focus on GFP+ cells located in the midbrain and in the rostral hindbrain. Dorsal views of single focal planes. Scale bar for all panels: (**A**) 100 µm. For better representation of low and high signal intensities, non-linear adjustments were made to whole image panels (see Methods Section 2.8). Abbreviations: AC - amacrine cells; DAC1-6 - dopaminergic cluster 1-6; Mb - midbrain; OB - olfactory bulb; Pr - pretectum; rHb - rostral hindbrain; Sp - subpallium.

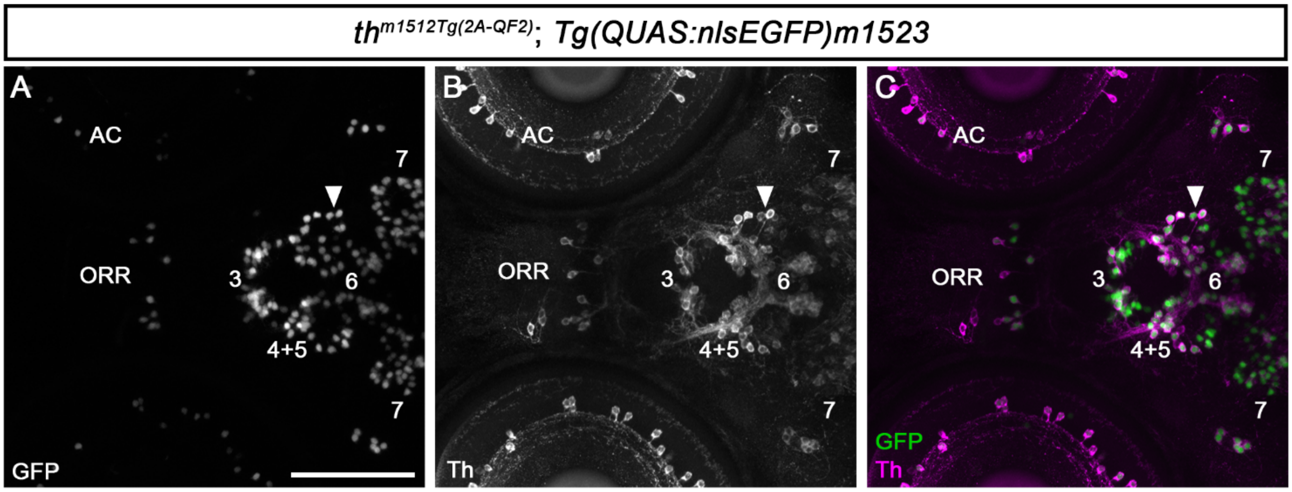

Supplementary Figure 2. Th expressing neurons in the posterior tuberculum and hypothalamus.

(A-C) Whole-mount immunofluorescence for GFP (green) and Th (magenta) of a *th* $^{m1512Tg(2A\text{-}QF2)}$; *Tg(QUAS:nlsEGFP)m1523* embryo at 96 hpf. Dorsal views of maximum intensity projections of a z-stack with 62 focal planes and z-step 1.18 µm. Anterior is to the left. Neurons expressing nuclear EGFP in the optic recess region, posterior tuberculum (DAC4), and hypothalamus (DAC3, 5-7) show a strong coincidence with Th expression. While the Th immunofluorescence in the DAC7 cells of the posterior recess region is weak compared to the nuclear GFP immunofluorescence, GFP+ cells are also Th+. Scale bar: 100 µm in A for all panels. For better representation of low and high signal intensities, non-linear adjustments were made to whole image panels (see Methods Section 2.8). Abbreviations: AC - amacrine cells; DAC - dopaminergic cluster (only numbers shown); ORR – optic recess region.

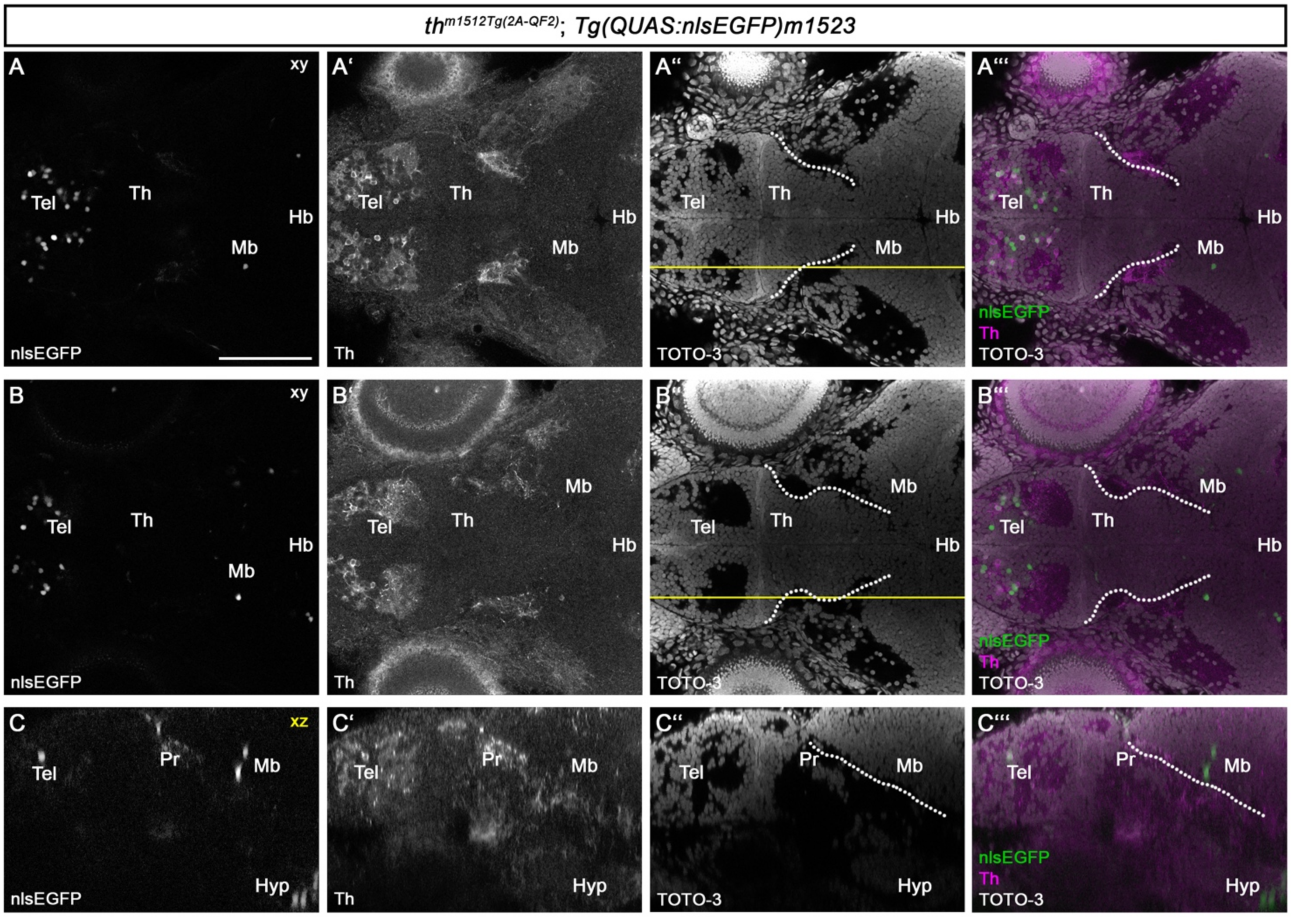

Supplementary Figure 3. Newly detected Th+ neurons reside in the midbrain and not in the diencephalon.

(A-C''') Whole-mount Immunofluorescence and nuclear staining for GFP (green), Th (magenta) and TOTO-3 (greyscale) of a *th $^{m1512Tg(2A-QF2)}$*; *Tg(QUAS:nlsEGFP)m1523* embryo at 96 hpf. Dorsal, xy, (**A-B'''**) views of single focal planes, and sagittal, xz, view (**C-C'''**). Anterior is to the left. The dotted lines mark the diencephalic-mesencephalic border. **(A-A''')** Dorsal view of nuclear EGFP expression in comparison to Th expression and nuclear TOTO-3 staining in the telencephalon and midbrain. **(B-B''')** More ventral view than panel A of the same stained embryo. Yellow lines in A''' and B''' indicate position of the sagittal view shown in C-C'''. **(C-C''')** Sagittal view of the embryo shown in A-B'''. Scale bar: 100 µm in a for all panels. For better representation of low and high signal intensities, non-linear adjustments were made to whole image panels (see Methods Section 2.8). Abbreviations: Hyp - hypothalamus; Mb - midbrain; Pr - pretectum; Tel - telencephalon; Th - thalamus.

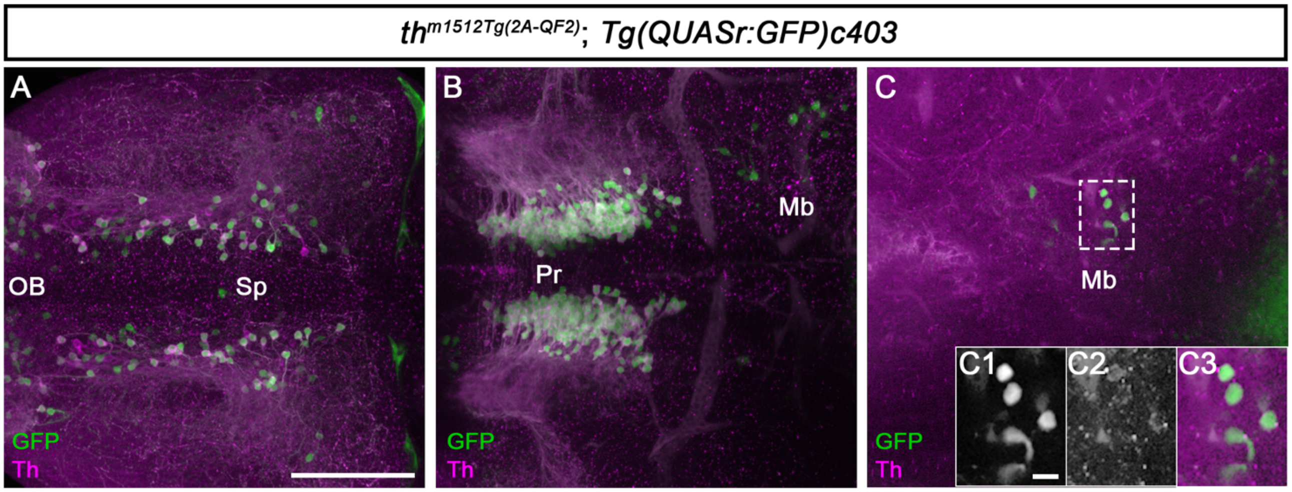

Supplementary Figure 4. Midbrain Th+ neurons persist into juvenile stages.

(A-C3) Whole-mount immunofluorescence staining for GFP (green) and Th (magenta) of a *th* $^{m1512Tg(2A-QF2)}$; *Tg(QUASr:GFP)c403* cleared brain at 30 dpf. Dorsal views of z-projections (**A, B**) or single planes (**C-C3**). Anterior is to the left. **(A)** GFP and Th expression in the subpallium and olfactory bulb. **(B)** Expression of GFP and Th, focusing on the pretectum and midbrain. **(C)** GFP+ cells in the midbrain marked by a dashed box. **(C1-C3)** Magnification of the cells marked in C showing GFP and Th expression. Scale bars: (**A** also for **B**, **C**) 100 µm; (**C1** also for **C2**, **C3**) 10 µm. For better representation of low and high signal intensities, non-linear adjustments were made to whole image panels (see Methods Section 2.8). Abbreviations: Mb – midbrain; OB – olfactory bulb; Pr – pretectum; Sp – subpallium.

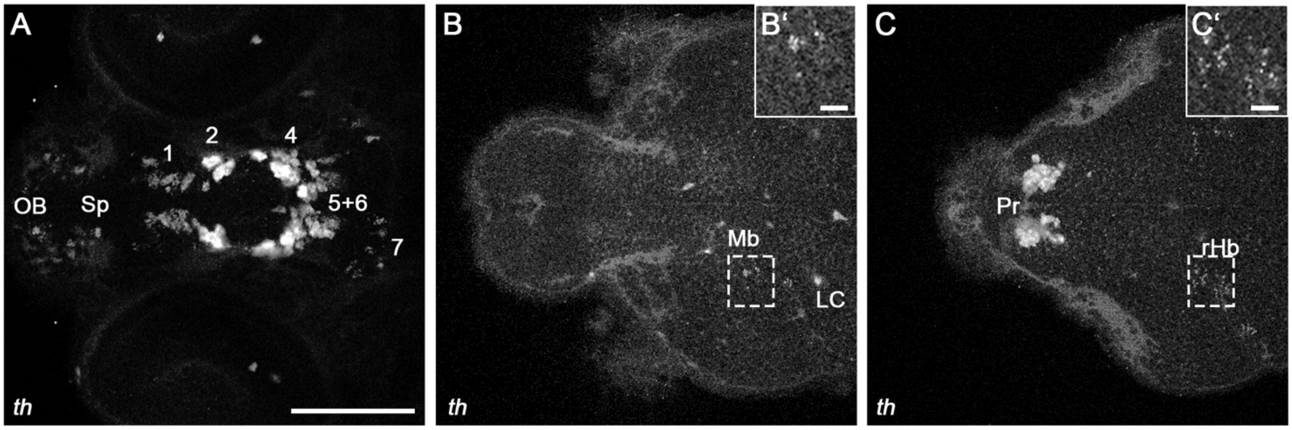

Supplementary Figure 5. Expression of *th* in the Mb and rHb cells is also detected in non-transgenic wildtype zebrafish.

(A-C') Whole-mount HCR RNA-FISH for *th* (grey) in an ABTL embryo at 96 hpf. Dorsal views of single planes. **(A)** Expression of *th* in the DA groups in the olfactory bulb, subpallium, and in the DAC1-7 groups. **(B)** Expression of *th* in the midbrain and in one neuron of the locus coeruleus. Dashed box indicates magnified area in B'. **(B')** Magnification of cells in B shows *th* transcripts in the midbrain. **(C)** Expression of *th* in the pretectal DA group and in the rostral hindbrain. Dashed box indicates magnified area in C'. **(C')** Magnification of cells in C shows *th* transcripts in the rostral hindbrain at the position of rHb GFP+ cells in $th^{m1512Tg(2A\text{-}QF2)}$; *Tg(QUASr:GFP)c403*. Scale bars: (**A** also for **B**, **C**) 100 µm; (**B'**,**C'**) 10 µm. For better representation of low and high signal intensities, non-linear adjustments were made to whole image panels (see Methods Section 2.8). Abbreviations: LC - locus coeruleus; Mb - midbrain; OB - olfactory bulb; Pr - pretectum; rHb - rostral hindbrain; Sp - subpallium.

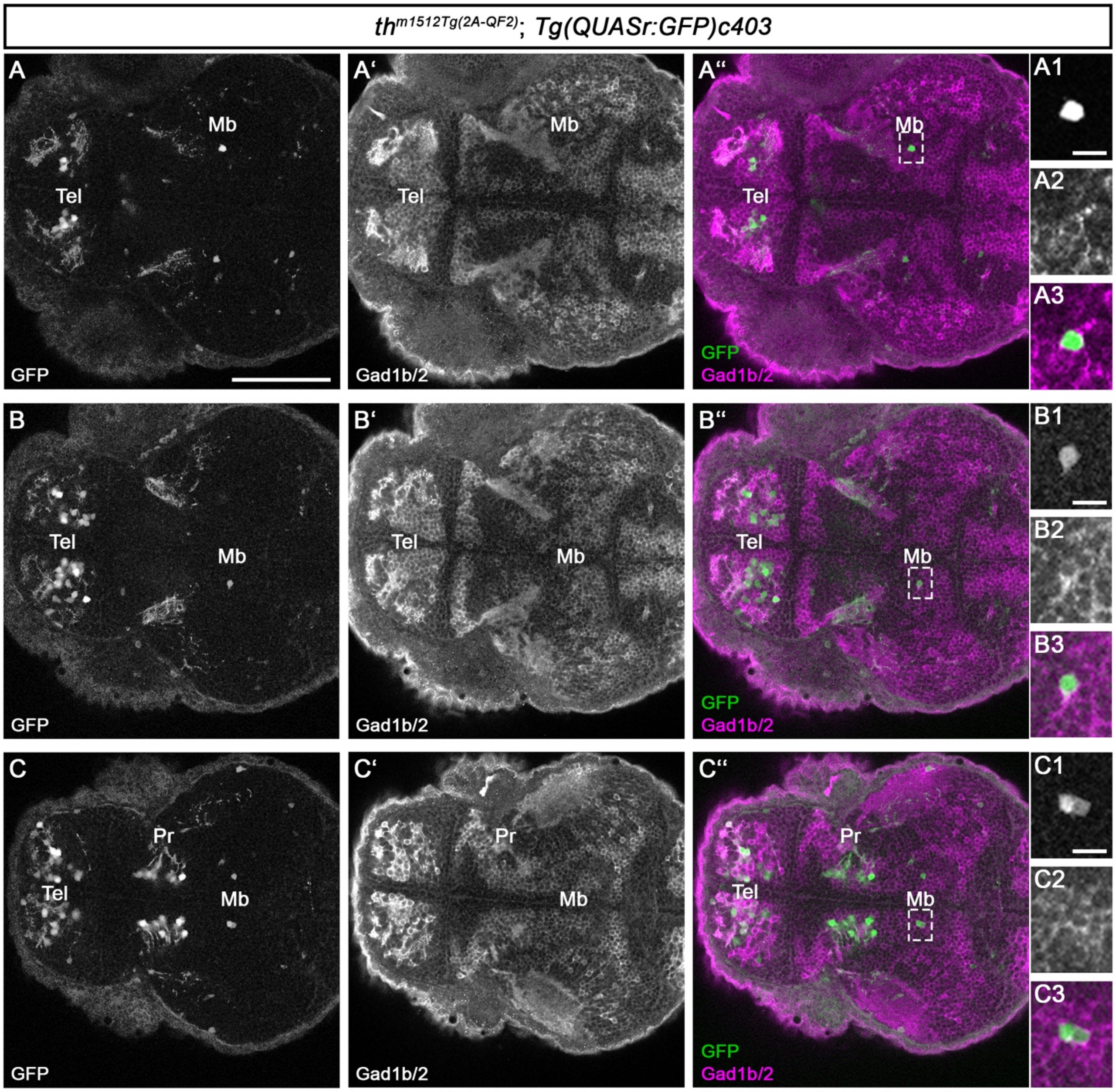

Supplementary Figure 6. Midbrain Th+ neurons are GABAergic.

(A-C3) Whole-mount immunofluorescence staining for GFP (green) and Gad1b/2 (magenta) of a *th* *m1512Tg(2A-QF2)*; *Tg(QUASr:GFP)c403* embryo at 96 hpf. Dorsal views of single planes. Anterior is to the left. **(A-A'')** Expression of GFP and Gad1b/2 in the telencephalon and midbrain. **(A1-A3)** Magnification of the GFP+ cell in the midbrain marked by a dashed box in A''. **(B-B'')** Expression of GFP and Gad1b/2 in the telencephalon and midbrain in a more dorsal view compared to A-A''. **(B1-B3)** Magnification of the GFP+ cell in the midbrain marked by a dashed box in B''. **(C-C'')** Expression of GFP and Gad1b/2 in the telencephalon, pretectum and midbrain. **(C1-C3)** Magnification of the GFP+ cell in the midbrain marked by a dashed box in C''. Total z-stack volume of 192 focal planes covering all DA neuronal groups of the forebrain and z-step of 1.18 µm with focal plane 1 being most ventral and focal plane 192 most dorsal. (**A**) focal plane 120. (**B**) focal plane 130 (**C**) focal plane 145.

Scale bars: (**A** for **A-C''**) 100 µm; (**A1**, **B1**, **C1**) 10 µm. For better representation of low and high signal intensities, non-linear adjustments were made to whole image panels (see Methods Section 2.8). Abbreviations: Mb - midbrain; Pr - pretectum; Tel - telencephalon.

4 Supplementary videos

Supplementary Video 1. GFP expression in vivo in *th* $^{m1512Tg(2A\text{-}QF2)}$; *Tg(QUASr:GFP)c403* larvae at 15 dpf.

In vivo 2-Photon recording of a *th* $^{m1512Tg(2A\text{-}QF2)}$; *Tg(QUASr:GFP)c403* larva at 15 dpf. The top panel is a stack of 314 slices with a z-step of 1.23 µm. The lower panel is a maximum intensity projection of a sagittal reslicing of the stack in the upper panel. The moving line indicates the progression of the video in correspondence to the sagittal view. Anterior is to the left. Scale bars: 100 µm.
